# Genome-wide analyses reveal intricate genetic mechanisms underlying egg production efficiency in chickens

**DOI:** 10.1186/s40104-025-01245-2

**Published:** 2025-08-11

**Authors:** Lizhi Tan, Xinyu Cai, Yuan Kong, Zexuan Liu, Zilong Wen, Lina Bu, Yuzhan Wang, Xiaojun Liu, Zhiwu Zhang, Jianlin Han, Dandan Wang, Yiqiang Zhao

**Affiliations:** 1 State Key Laboratory of Animal Biotech Breeding, College of Biological Sciences, China Agricultural University, Beijing, 100193 China; 2 College of Animal Science and Technology, China Agricultural University, Beijing, 100193 China; 3 National Research Facility for Phenotypic and Genotypic Analysis of Model Animals (Beijing), China Agricultural University, Beijing, 100193 China; 4 College of Animal Science and Technology, Henan Agricultural University, Zhengzhou, 450046 China; 5 Department of Crop and Soil Sciences, Washington State University, Pullman, WA USA; 6 Yazhouwan National Laboratory, Sanya, 572024 China; 7 College of Animal Science and Veterinary Medicine, Henan Institute of Science and Technology, Xinxiang, 453003 China

**Keywords:** Egg production efficiency, Genome-wide association study, Polygenic architecture, Polygenic selection

## Abstract

**Background:**

Compared to many other vertebrates, chickens have a high reproductive efficiency in terms of egg production. The classic traits for evaluating egg-laying performance include age at first egg, egg number, clutch size, laying rate, etc. These egg-laying traits were not specifically designed to characterize egg production efficiency and stability. By considering the stage-specific variations in the egg production curve, this study aims to investigate the genetic mechanisms that directly influence the efficiency of egg production at each stage of the laying cycle.

**Results:**

Using whole-genome sequencing data, we perform comprehensive genome-wide association study for 39 traits that focus on egg production efficiency and stability in the Gushi chicken. We showed that the haplotype-based approach is more effective for genetic mapping and capturing polygenic architecture. By combining the signals of Singleton Density Score (SDS), which is a population-genetic statistic designed to detect recent selection by leveraging the distribution of singletons, and association analyses, multiple egg-laying traits related to egg production efficiency were found to have experienced polygenic selection. Consistently, functional analysis of associated genes demonstrates that egg production efficiency benefits from multiple physiological functions. Furthermore, our results identified the *CNNM2* gene, known for its role in magnesium homeostasis, plays a dual role in egg production variance, promoting variability during the up-stage while reducing it during the sustained-stage to optimize egg production efficiency.

**Conclusions:**

Collectively, our multiple genome analyses reveal a complex genetic mechanism underlying more efficient and stable egg production, and establish chicken genetics as a model for studying reproductive efficiency across species.

**Supplementary Information:**

The online version contains supplementary material available at 10.1186/s40104-025-01245-2.

## Background

Egg production in chickens is primarily controlled and regulated by the hypothalamic-pituitary-ovary (HPO) axis which govern the dynamics of ovulation, egg formation, and oviposition [1]. To understand the genetic basis of egg production, traditional egg-laying traits such as age at first egg, egg number, clutch size and laying rate, were widely investigated by quantitative trait loci (QTL) mapping and genome-wide association study (GWAS) [2–5]. A large number of genetic variants have been identified as significant contributors to these traits, with the goal of enhancing egg production [6]. Egg production process is intricate and multifaceted. Beyond identifying key variants and associated genes, our recent study shows that the inter-tissue crosstalk of endocrine factors with the HPO axis plays an indispensable role in the indirect regulation of egg production [7].

Egg production throughout a hen's laying cycle can be characterized by egg production curves, which typically encompass stages from onset to peak, sustained production, and eventual decline [8]. From the perspective of the curve, a viable strategy to enhance egg production involves optimizing performance at each stage of the laying cycle [9]. This includes accelerating the rapid increase to peak production, maintaining peak levels to delay decline, thereby improving overall egg production while extending the laying cycle. Despite considerable efforts, the intricate genetic mechanisms underlying egg production remain poorly understood from these perspectives. In addition, enhancing egg production requires thorough investigation of the genetic architecture of egg-laying traits, including their effect sizes, polygenicity and possible pleiotropy [10]. A comprehensive study of genetic basis of complex egg-laying traits is therefore of great importance for deciphering gene-trait relationship and making informed decisions towards advancing breeding strategies [5, 11].

In this work, we extended our previous study on the genetic mechanisms underlying egg production by investigating 39 newly derived traits that focus on egg production efficiency and stability. Egg production stability here refers to reduced variance in production traits and consistent performance, while egg production efficiency in this study is defined as the hen’s ability to produce a high number of eggs consistently over the entire laying cycle. We consider egg production efficiency to encompasses two key dimensions: (1) a high effective clutch intensity (ECI), which indicates strong laying persistence during a clutch period, and (2) specific patterns in the laying curve, characterized by a rapid increase to peak production during the up-stage, followed by a prolonged period of stable egg production during the sustained-stage. Through comprehensive genome-wide association studies, we identified novel genetic signals and functionally relevant associated genes, and effectively elucidated the polygenic structure of egg-laying traits. We found that egg production variance traits experienced positive selection during the up-stage of laying, while egg production variance was selected against during the sustained-stage. Consistent with this principle, canonical correlation-based association analysis identified magnesium transporter gene *CNNM2* (Cyclin and CBS Domain Divalent Metal Cation Transport Mediator 2) to play a dual role on egg production variance by promoting the variance in the up-stage while decreasing the variance in the sustained-stage. Together, these findings reveal an intricate genetic mechanism that promotes more efficient and stable egg production. Our research advances the understanding of avian reproductive biology and provides valuable insights for improving egg production in chickens.

## Materials and methods

### Ethics statement

All animal experimental protocols were approved by the Institutional Animal Care and Use Committee of Henan Agricultural University (protocol number 11-0085). The methods were carried out in accordance with the approved guidelines.

### Sequencing and genotyping

For the resequencing data of 900 chickens, the reads obtained are of high quality with an average sequencing depth of 5.5 × [7]. The highest-quality mapping reads were calibrated using fastp with default parameters [12]. The reads were mapped against the chicken reference genome GRCg6a (GenBank accession No. GCF_000002315.6) using GTX-align with default parameters, a commercial FPGA-accelerated version of BWA [13]. Mapped reads were sorted and de-duplicated using SAMtools (V.1.3.167) [14].

The BaseVar-STITCH pipeline was used to identify polymorphic loci and impute genotypes from the mapped bam files [15]. The BaseVar-STITCH pipeline consists of two steps: step 1, variant calling with BaseVar: “python BaseVar.py basetype -R Gallus6.fa --regions chr --align-file-list bam_list --output-vcf chr_vcf.gz -output-cvg chr_cvg.tsv.gz --nCPU 6 --filename-has-samplename --smart-rerun”; step 2, genotype imputation with STITCH: “Rscript STITCH.R --chr 1 --bamlist bam_list -reference Gallus6.fa --K 28 --nGen 12 --nCores 20 --niterations 40 --outputdir chr1 --tempdir tmp1 --posfile chr1_ posfile”. The steps are the same as in our previous work [7]. PLINK (V1.90) [16] software was then used to screen for sample quality by setting the sample call rate (> 97%) and filter out low-quality SNPs, including SNPs with a call rate < 98%, minor allele frequency (MAF) < 0.02, and Hardy–Weinberg equilibrium *P* < 1 × 10^–6^. Finally, 888 individuals and 13,494,226 autosomal SNPs were qualified for subsequent analysis. All qualified SNPs were pruned using the parameter --indep-pairwise 50 10 0.6 in PLINK (V1.90) to obtain 3,616,394 independent SNPs for SNP-based GWAS and CCA-based GWAS analyses.

Haplotype phasing were performed using Beagle (V5.0) [17] with the parameters: beagle.jar gt = file.vcf out = file.phased gp = true. For phased data, 7,065,827 SNPs were obtained by using the parameter --indep-pairwise 50 10 0.98 in PLINK. The prune threshold of 0.98 was chosen to filter out completely linked SNPs. The steps are the same as in our previous work [7]

### Imputation of missing original egg number

A total of 888 Gushi chickens were used for the GWAS of egg-laying traits. The 12^th^ generation Gushi hens were obtained from the core breeding population of the Gushi Chicken Breeding Farm at Henan Sangao Agriculture and Animal Husbandry Co., Ltd., Henan Province, China. The population consisted of two batches of chickens, raised until 12 weeks of age, and then transferred to single cages in the same coop. Egg numbers were recorded daily until 43 weeks of age. Missing data from 21 to 43 weeks were imputed using the Random Forest method, implemented in R package ‘randomForest' using parameters: ntree = 200, maxiter = 10, mtry = 10. The Random Forest method uses the complete data as the training set. It begins by imputing average values for missing data, starting with columns that have the fewest missing data points. The process then iterated until the parameters converged (Supplementary Fig. S14).

### Egg production rate curve and derived egg-laying traits

In contrast to the conventionally delineated egg-laying period used by Wang et al. [7] we introduced a curve-fitting strategy to explore the dynamic egg-laying pattern. Three egg production rate models (Wood model [18], compartmental model [19], and Yang-Ning model [20]) were employed to fit the average egg production rate curve based on individual egg production data. The model expressions are as follows:

Wood model: $$y_t=at^be^{-ct}$$

compartmental model: $$y_t=a{(1-e}^{-c(t-d)})e^{-bt}$$

Yang-Ning model: $y_t=ae^{-bt}/(1+e^{-c\left(t-d\right)})$

Where *t* is the week of age, ${{y}}_{{t}}$ is the laying rate at week *t*, $a$, $b$, $c$ and $d$ are the parameters to be measured, respectively. In the Yang-Ning model, $a$ denotes the maximum potential of egg production rate, $b$ denotes the rate of decline, $c$ denotes the rate of increase, and $d$ denotes the week of age at first egg.

According to the fitted curve, the egg production process was divided into three stages: up-stage (21–26 weeks), sustained-stage (27–43 weeks), and all-stage (21–43 weeks). Based on the daily egg number records, we generated a serial of newly derived traits to characterize of the egg-laying feature compared to our previous study. They are 5 production traits, including egg production variance (EV), weekly maximum laying rate (WMLR), weekly egg production variance (WEV), bi-weekly maximum laying rate (BWMLR) and bi-weekly egg production variance (BWEV). Three traits related to clutch, including clutch period number (CPN), total clutch size (TCS), and effective clutch intensity (ECI). Five traits related to interval, including laying interval time (LIT), average inter-laying interval (AILI), total inter-laying interval (TILI), maximum inter-laying interval (MILI), and interclutch interval (II). Supplementary Table S1 describes the definition and descriptive statistics of each derived trait. For each derived trait, a 3σ criteria was applied to remove outlier individuals to ensure the accuracy of the subsequent GWAS analyses.

The complex derived traits ECI (defined as TCS divided by CPN) and II (defined as the number of intervals divided by CPN) are calculated under the condition that each clutch includes at least two consecutive laying days:

$$ECI=\frac{{TCS}_{c\geq2}}{{CPN}_{c\geq2}}$$

$$II=\frac{\sum_{t_2}^{t_1}i}{{CPN}_{c\geq2}}$$

where ${t}_{1}$ denotes the start of the laying age (21 weeks), ${t}_{2}$ denotes the end of the laying age (43 weeks); ${c}$ represents the length of clutch in a clutch period; $\textit{i}$ represents the number of interval time in a clutch period [21].

### SNP-based GWAS

Population stratification is one of the major causes for spurious phenotype-genotype associations in GWAS analysis. The projection on PC1 and PC2 showed a high overlap of individuals originating from two batches (Supplementary Fig. S15), suggesting that population stratification between batches was largely absent. Therefore, individuals from different batches were combined in the subsequent analyses. To err on the side of caution, we included the first 10 principal components (PCs) as covariates in the SNP-based GWAS analysis to control for possible confounds. The genomic relationship matrix (GRM) is a matrix that represents genetic similarity between individuals, calculated using genomic markers. It helps control for false positives by modeling the random effects of polygenic background.

After genotype imputation and quality control, 3,616,394 SNPs were obtained genome-wide. SNP-based GWAS analysis was performed for the 39 traits for the population of 843–888 chickens. SNP-based GWAS for egg-laying traits was conducted using fastGWA method [22] implemented in GCTA (V1.94.1), under the mixed linear model:

$${\varvec{y}}={\varvec{Q}}\boldsymbol{\alpha }+{\varvec{x}}{\varvec{\beta}}+{\varvec{g}}+{\varvec{e}}$$

where ${\varvec{y}}$ is the vector of egg-laying trait, ${\varvec{Q}}$ is the design matrix of covariates, including the top 10 PCs calculated from genome-wide SNPs by GCTA (V1.94.1), and hatch batch to correct population stratification; ${\varvec{x}}$ is the vector of genotype encoded by 0, 1, or 2; ${\varvec{g}}$ is the polygenic effect captured by GRM calculated from genome-wide SNPs; $\boldsymbol{\alpha }$ and $\boldsymbol{\beta}$ are the vectors of corresponding effect size; and ${\varvec{e}}$ is the residual. Genome-wide significant *P* values were corrected by FDR (false discovery rate) $\le$ 0.05. The Manhattan and Q-Q plots of egg-laying traits were plotted using the R package ‘ggplot2' and ‘CMplot' [23]. LD of GWAS signal interval was presented by LDBlockShow software (V1.40) [24].

### Haplotype-based GWAS

The genome was divided into 1,413,153 blocks of five successive SNPs. Haplotypes within a block were retrieved and each diploid individual was coded by combination of two haplotype alleles. Haplotype-based GWAS analysis was performed using the R package ‘lme4qtl' [25]. The model can be written as:

$${\varvec{y}}={{\varvec{Q}}\boldsymbol{\alpha }+{\varvec{x}}}_{{\varvec{h}}}{\varvec{\beta}}+{{\varvec{g}}}_{{\varvec{h}}}+{\varvec{e}}$$

where ${\varvec{y}}$ is the vector of egg-laying trait, ${\varvec{Q}}$ is the design matrix of covariates, including the birth batch; ${{\varvec{x}}}_{{\varvec{h}}}$ is the haplotype combination as categorical variable; ${{\varvec{g}}}_{{\varvec{h}}}$ is the polygenic effect captured by GRM calculated from genome-wide haplotypes; $\boldsymbol{\alpha }$ is the effect for the covariates; ${\varvec{\beta}}$ is the fixed effect of haplotype combination to be tested for association; and ${\varvec{e}}$ is the residual. To enhance computational efficiency, we first constructed a null model excluding haplotype combination effects (incorporating only haplotype GRM random effects) using lme4qtl. This null model was established once in advance. Subsequently, the model residual was used as new phenotypes to build simple linear models. These models were used to test the overall statistical significance of each haplotype block.

The haplotype-based GRM was calculated with reference to method 1 described by Ferdosi et al. [26]. For each block *i*, we assigned a value of 1 when two haplotype alleles are equal and 0 when they are not, to obtain the haplotype relationship matrix ${\Gamma }_{i}$. The genome-wide haplotype matrix $\boldsymbol\Gamma$ is obtained as: $\boldsymbol\Gamma=\sum_{i=1}^n{\boldsymbol\Gamma}_i/n$, where *n* is the number of haplotype blocks in the genome. We finally converted the haplotype relationship matrix into an individual-level relationship matrix using: $\boldsymbol{H}=\boldsymbol{K}\boldsymbol{\Gamma} \boldsymbol{K}^{\prime}/2$, where $\boldsymbol{K }=\boldsymbol{ I }\otimes [1 1]$ (***I*** is the identity matrix of $\mathit{m} \times \mathit{m}$, where $\mathit{m}$ is the number of individuals and $\otimes$ is the Kronecker product).

### CCA-based GWAS

Canonical correlation analysis (CCA) uses linear combinations of variables derived from two sets of data objects to find the combination that is maximally correlated. In our study, the CCA-based GWAS used the same set of SNPs as used in the SNP-based GWAS but extended the association analysis of a single trait to multiple traits. The model can be written as:

$$\rho ({{\varvec{x}}}^{\boldsymbol{^{\prime}}},{{\varvec{y}}}^{\boldsymbol{^{\prime}}})=\frac{Cov({{\varvec{x}}}^{\boldsymbol{^{\prime}}},{{\varvec{y}}}^{\boldsymbol{^{\prime}}})}{\sqrt{Var\left({{\varvec{x}}}^{\boldsymbol{^{\prime}}}\right)}\sqrt{Var({{\varvec{y}}}^{\boldsymbol{^{\prime}}})}}$$

where $\rho ({{\varvec{x}}}^{\boldsymbol{^{\prime}}},{{\varvec{y}}}^{\boldsymbol{^{\prime}}})$ is the correlation of ${{\varvec{x}}}^{\boldsymbol{^{\prime}}}$ and ${{\varvec{y}}}^{\boldsymbol{^{\prime}}}$ projection vectors; ${\varvec{y}}$ is the vector of multiple egg-laying trait from the same laying stage (up-stage, sustained-stage, or all-stage); ${{\varvec{y}}}^{\boldsymbol{^{\prime}}}$ is the projection vector of ${\varvec{y}}$, denotes ${{\varvec{b}}}^{{\varvec{T}}}{\varvec{y}}$. ${\varvec{x}}$ is the vector of genotype encoded by 0, 1, or 2; ${{\varvec{x}}}^{\boldsymbol{^{\prime}}}$ is the projection vector of ***x***, denotes ${{\varvec{a}}}^{{\varvec{T}}}{\varvec{x}}$; ${\varvec{a}}$ and ${\varvec{b}}$ maximize $\rho ({{\varvec{x}}}^{\boldsymbol{^{\prime}}},{{\varvec{y}}}^{\boldsymbol{^{\prime}}})$ to obtain the corresponding projection vector.

For each SNP, a *P-*value was estimated by calculating the correlation between genotype and multiple traits using the Chi-square test. The CCA-based GWAS also reports the canonical correlation coefficients between genotype and trait value, which can be used to measure the relative contribution of SNPs to specific traits. CCA is implemented using the ‘cancor' function in the R package ‘stats'.

### Genome-wide prediction using haplotypes

Before estimating the effect size of haplotype alleles, we implemented a numerical dosage coding strategy. For each significant haplotype block identified by HGWAS, diploid individuals were coded by 0, 1, or 2, representing the number of copies of a specific haplotype allele present in the respective individual. The sum of codes across all haplotype alleles within a block equal two for each individual.

Two haplotype-based phenotype prediction methods are proposed. In the first method, the effect size of haplotype alleles was estimated as fixed effects. The model can be written as:

$${\varvec{y}}={\varvec{Q}}\boldsymbol{\alpha }+{\varvec{x}}{\varvec{\beta}}+{\varvec{e}}$$

where ${\varvec{y}}$ is the vector of egg-laying trait; ${\varvec{Q}}$ is the design matrix of covariates, including the birth batch; ${\varvec{x}}$ is the indicator of haplotypes dosage code; $\boldsymbol{\alpha }$ is the effect vector of covariates; ${\varvec{\beta}}$ is the vector of effect size of haplotype allele as fixed effect; and ${\varvec{e}}$ is the residual. For haplotype alleles showing statistical significance (*P*-value < 0.05), those with a positive effect size were classified as BHA, while those with a negative effect size were classified as UHA. After obtaining the effect sizes of haplotype alleles, the haplotype-based polygenic prediction score (HPPS) was calculated by summing the effects of multiple haplotype alleles from all significant blocks to estimate an individual's phenotypic predictions. We calculated three varieties: HPPS_all for all significant haplotype blocks, HPPS_bene for only blocks comprising BHA, and HPPS_unbe for only blocks comprising UHA. The HPPS calculations were performed using the lm function in R.

The second method is very similar to the first one, except that the effect of haplotype alleles was estimated as the random effects. The model can be written as:

$${\varvec{y}}={\varvec{Q}}\boldsymbol{\alpha }+{\varvec{Z}}{\varvec{\mu}}+{\varvec{e}}$$

where ${\varvec{Z}}$ is the indicator of haplotypes dosage code; ${\varvec{\mu}}$ is the vector of effect of haplotype allele as random effect, and ${\varvec{e}}$ is the residual. For random effects, we used random effect estimates for all haplotype alleles and called the prediction as Haplotype-based Best Linear Unbiased Prediction (HBLUP). The HBLUP calculations were performed using the R package ‘hglm'.

To evaluate the accuracy of genomic prediction, we implemented a constant validation set approach, wherein a fixed subset of individuals was consistently excluded from model training. Specifically, we randomly selected 100 individuals to constitute a fixed validation set. The model's accuracy was then evaluated exclusively using this constant validation set within the 10-fold cross-validation (CV) framework. The Pearson correlation coefficient was used to quantify the correlation between predicted and observed phenotypes. This process was repeated 50 times, and we reported the mean correlation coefficient as the measure of prediction accuracy.

### Analysis on allele-stage interactions

To investigate the interaction between genotype and stages on egg-laying trait, we employed GLM with an interaction term. In this model, the up-stage and sustained-stage of egg production were treated as one fixed effect, while the genotypes were considered as another fixed effect. The model can be written as:

$${\varvec{y}}={\varvec{Q}}\boldsymbol{\alpha }+{\varvec{x}}{\varvec{\beta}}+{\varvec{\gamma}}\left({\varvec{Q}}\times {\varvec{x}}\right)+{\varvec{e}}$$

where ${\varvec{y}}$ is the vector of egg-laying trait; ${\varvec{Q}}$ is the vector of stage as factors (up-stage/sustained-stage); ${\varvec{x}}$ is the vector of genotype as factors (aa/Aa/AA); $\boldsymbol{\alpha }$ is the effect vector of stage; ${\varvec{\beta}}$ is the effect vector of each SNP; ${\varvec{\gamma}}$ is the interaction effect between stage and genotype; and ${\varvec{e}}$ is the residual. A post-hoc analysis was subsequently conducted to identify the specific level of interaction between stage and genotype, when ${\varvec{\gamma}}$ tests significant. The post-hoc test was implemented by the R package ‘emmeans'.

### Proportion of variance explained by genetic loci

The proportion of variance explained (PVE) by SNP or haplotype was calculated following the method proposed by Gu et al. [27]. A random model was used to fit both significant loci and insignificant loci. The model can be written as:

$${\varvec{y}}={{\varvec{Z}}}_{1}{{\varvec{\mu}}}_{1}+{{\varvec{Z}}}_{2}{{\varvec{\mu}}}_{2}+{\varvec{e}}$$

where ${\varvec{y}}$ is the vector of egg-laying trait; ${{\varvec{Z}}}_{1}$ and ${{\varvec{Z}}}_{2}$ are design matrixes of genotype encoded by 0, 1, or 2; ${{\varvec{\mu}}}_{1}$ is the vector of the genetic effect for significant loci, and ${{\varvec{\mu}}}_{2}$ is the vector of the genetic effect for insignificant loci, with ${\text{Var}({\varvec{\mu}}}_{i})\sim \text{N}(0,\ {{\varvec{K}}}_{{\varvec{i}}}{\sigma }_{{{\varvec{\mu}}}_{{\varvec{i}}}}^{2})$, where ${{\varvec{K}}}_{{\varvec{i}}}$ denotes the GRM constructed from the significant loci (*i* = 1) and insignificant loci (*i* = 2), and ${\sigma }_{{{\varvec{\mu}}}_{{\varvec{i}}}}^{2}$ denotes the genetic variance corresponding to the random effect; and ${\varvec{e}}$ is the residual. The PVE of significant loci from SNP-based GWAS or haplotype-based GWAS can be expressed as $\frac{{\sigma }_{{{\varvec{\mu}}}_{1}}^{2}}{{\sigma }_{{{\varvec{\mu}}}_{1}}^{2}+ {\sigma }_{{{\varvec{\mu}}}_{2}}^{2}+ {\sigma }_{e}^{2}}$. The genome-wide PVE from SNP-based GWAS and haplotype-based GWAS can be expressed as $\frac{{\sigma }_{{{\varvec{\mu}}}_{1}}^{2}+ {\sigma }_{{{\varvec{\mu}}}_{2}}^{2}}{{\sigma }_{{{\varvec{\mu}}}_{1}}^{2}+ {\sigma }_{{{\varvec{\mu}}}_{2}}^{2}+ {\sigma }_{e}^{2}}$. The variance components were estimated by the EMREML approach using “Single trait model” from HIBLUP (V1.4) [28].

### Enriched score for beneficial and unbeneficial haplotype allele in Chinese chicken populations

For significant haplotype blocks identified by HGWAS across different traits, we traced the origin and examined the frequency of both BHA and UHA in 39 local chicken populations in China. Due to discrepancies between the two SNP sets, we filled missing loci in the 39 local chicken populations with the allele from the reference genome to make the haplotype alleles directly comparable. We defined an enriched score to measure the extent of enrichment of BHA and UHA in local chicken populations as:

$${E}_{k}= \sum_{i= 1}^{n}{f}_{nk}$$

where ${E}_{k}$ denotes the overall enriched score of a trait in the $\mathit k$^th^ population; $n$ denotes the number of BHA and UHA for this trait; and ${f}_{nk}$ denotes the frequency of the $n$^th^ haplotype allele in the $\mathit k$^th^ population.

### Historical effective population size estimation

Historical effective population size (Ne) was estimated from genome-wide SNPs by GONe software V1.0 using default parameters [29]. We ran GONe assuming a recombination rate of 300 kb per centimorgan and a generation time of one year using all 888 samples. As recommend, the GONe estimation is most reliable from recent 100–200 generations.

### Calculation of iHS, Tajima's D and π values

The integrated haplotype score (iHS) is a haplotype-based method for detecting positive selection, specifically focusing on strong hard sweeps. The iHS was calculated for the focal SNP by considering the extended haplotypes surrounding it, using the ihsbin program in hapbin software V1.3.0 [30] with the parameters: --minmaf 0.01 and --cutoff 0.01. The iHS values were Z-score standardized and then converted into *P*-values using the standard normal distribution. We applied Bonferroni correction at the genome-wide level to adjust these *P*-values.

We divided the genome into 100 K non-overlapped windows. The Tajima's D and π (nucleotide diversity) values were calculated for each window using VCFtools v0.1.17 [31] with the parameter -TajimaD 100000 and -window-pi 100000, respectively.

### SDS for recent polygenic selection

The singleton density score (SDS) is a statistical method for detecting recent selection based on the density of singletons. Under the assumption that haplotypes carrying the selected allele tend to carry fewer singleton mutations, this method models the changes in genealogical tip-branch lengths, calculated from distance between the nearest singletons between the derived and ancestral alleles.

We calculated SDS using the script provided by Field et al. [32]. The recent Ne was set to 20,436 according to the GONe estimation. We estimated gamma-shapes for each derived allele frequency (DAF) bin, with a bin width of 0.005 and DAF ranging from 0.05 to 0.95. As no significant difference was found between ancestral alleles (AAs) and derived alleles (DAs) in SDS calculation, we compared reference alleles with alternative alleles instead. Thus, SNPs with SDS > 0 indicate that the alternative allele was positively selected, and vice versa [33]. The raw SDS scores (rSDS) were converted to *P*-values using the Z-score approach, similar to the method applied for iHS. We identified potential selection regions based on high Z-scores of mean SDS values (mSDS) in non-overlapping genomic window of 50 kb.

### Associating tSDS with GWAS summary statistics

Trait-SDS (tSDS) were generated by polarizing the SDS scores such that a positive tSDS indicates an increased frequency of the allele favoring the trait. All SNPs with assigned tSDS were ranked by their GWAS −log_10_(*P*) in ascending order and grouped into consecutive bins of 1,000 SNPs each. For each of the 39 egg-laying traits, we calculated Spearman correlation coefficients between the rank of the bins and the average tSDS within each bin. A positive correlation suggests that the polygenic trait was favored by selection, while a negative correlation indicates not favored by selection.

### Multi-tissue transcriptome profiling

A total of 78 RNA sequencing samples (14 from the hypothalamus and 16 from pituitary, ovary, liver, and abdominal fat tissue) from 43-week-old high-yielding (*n* = 8) and low-yielding (*n* = 8) chickens were used retrieved from Wang et al. [7]. The multi-tissue transcriptome profiling using HISAT2 [34] and StringTie [35] are the same as in our previous work [7].

### Gene annotation, functional analysis and prioritization

To identify candidate genes that may affect chicken egg production, genes overlapping with GWAS significant loci were extracted according to the Ensembl genome annotation. Chicken QTL information from Chicken QTLdb (https://www.animalgenome.org/cgi-bin/QTLdb/GG/index) was used to match the candidate genes functionally related to egg-laying traits. GO enrichment analysis was performed using the Metascape web service (https://metascape.org) [36]. Due to data availability constraints, the enrichment analysis was based on chicken gene orthologs in mouse species.

Gene prioritization of candidate genes based on functional similarity was conducted using the ToppGene web service (https://toppgene.cchmc.org/) [37]. A total of 291 genes associated with human reproduction from the GWAS catalog (https://www.ebi.ac.uk/gwas/) [38] were used as the training set, and genes retrieved from SNP-based GWAS and haplotype-based GWAS were used as candidates to prioritize.

To reveal the biological context of the identified loci, a comprehensive biological network was constructed for *CNNM2* and the top 20 candidate genes using the web version of the GeneMANIA (https://genemania.org/) [39]. GeneMANIA integrates data on protein-genetic interactions, pathways, co-expression, co-localization, and protein structural domain similarity to construct biological networks and identify related genes in common pathways. The resulting networks were visualized using Cytoscape (V3.8.2) [40].

## Results

### Generating derived egg-laying traits

**Fig. 1:**
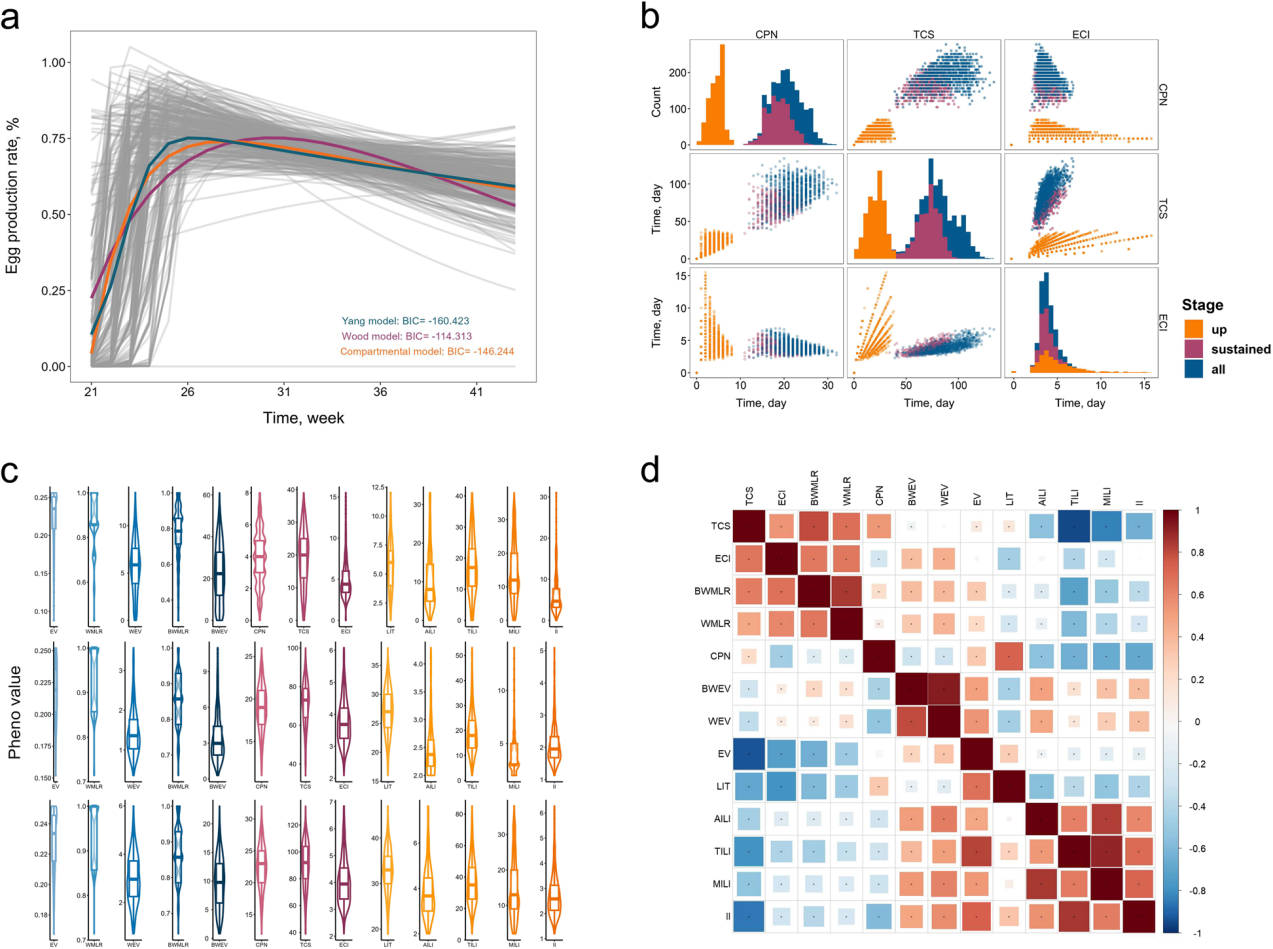
Trait generations and characteristics. **a** Three egg production rate models fitted to the average egg production rate of chickens. The grey folded line showing the observed egg production rate of each 888 individuals for 21–43 weeks. The blue curve is the fitted Yang-Ning model, the purple curve is the fitted Wood model, and the orange curve is the fitted compartmental model. The lower right corner shows the corresponding BIC values for each model. **b** Trait distribution of three clutch traits at three stages. Diagonal lines indicate single trait distributions, and upper and lower indicate scatter plots between two traits. The three colors indicate different stages. **c** Distribution of phenotype values. Each line represents a different stage, i.e., up-stage, sustained-stage, all-stage respectively. **d** Heatmap of Pearson correlation coefficients among 13 derived traits in the up-stage and sustained-stage. Upper triangle is for up-stage and lower triangle is for sustained-stage. Red color indicates coefficients > 0, blue indicates coefficients < 0, *P < 0.05

We considered the egg-laying behavior of chickens as a continuous and dynamic process from the start of egg-laying to the end of production. Multiple models were employed to fit the mean of the egg production rate, including the Wood model (BIC = −114.313), the compartmental model (BIC = −146.244), and the Yang-Ning model (BIC = −160.423) (Fig. 1a). The Yang-Ning model provides the best fit for the data, with its four parameters of specific biological implications: the maximum potential of egg production rate (a = 0.837), the rate of decline in egg production (b = 0.015), the rate of increase in egg production (c = 1.182), and the week of age at first egg (d = 2.621), altogether providing a plausible characterization of the biological features of the egg production.

According to the fitted curve of egg production rate, the stationary point of the mean curve in the chicken population was found to be at 26 weeks of age (${f}{\prime}\left(26.3\right)=0$). We, therefore, divided the egg-laying process into three stages: the up-stage (21–26 weeks), the sustained-stage (27–43 weeks), and the all-stage (21–43 weeks). Classifying these stages helps calibrate our understanding of the dynamics and underlying mechanisms of egg production throughout the laying cycle. Subsequent data analyses were conducted separately for the up-, sustained-, and all-stages. For the imputed egg production data, 13 derived traits related to production (5 traits), clutch (3 traits), and interval (5 traits) were constructed focusing on egg production efficiency and stability from the three egg-laying stages, totaling 39 traits (Fig. 1b, c, Supplementary Fig. S1, and Supplementary Table S1). Although some traits exhibited moderate to high phenotypic correlations (Fig. 1d and Supplementary Fig. S1), this does not necessarily indicate strong genetic correlation. Moreover, each trait has distinct biological significance. Therefore, all derived traits at each stage were retained for subsequent analyses.

### Genetic mapping for egg production efficiency

**Fig. 2:**
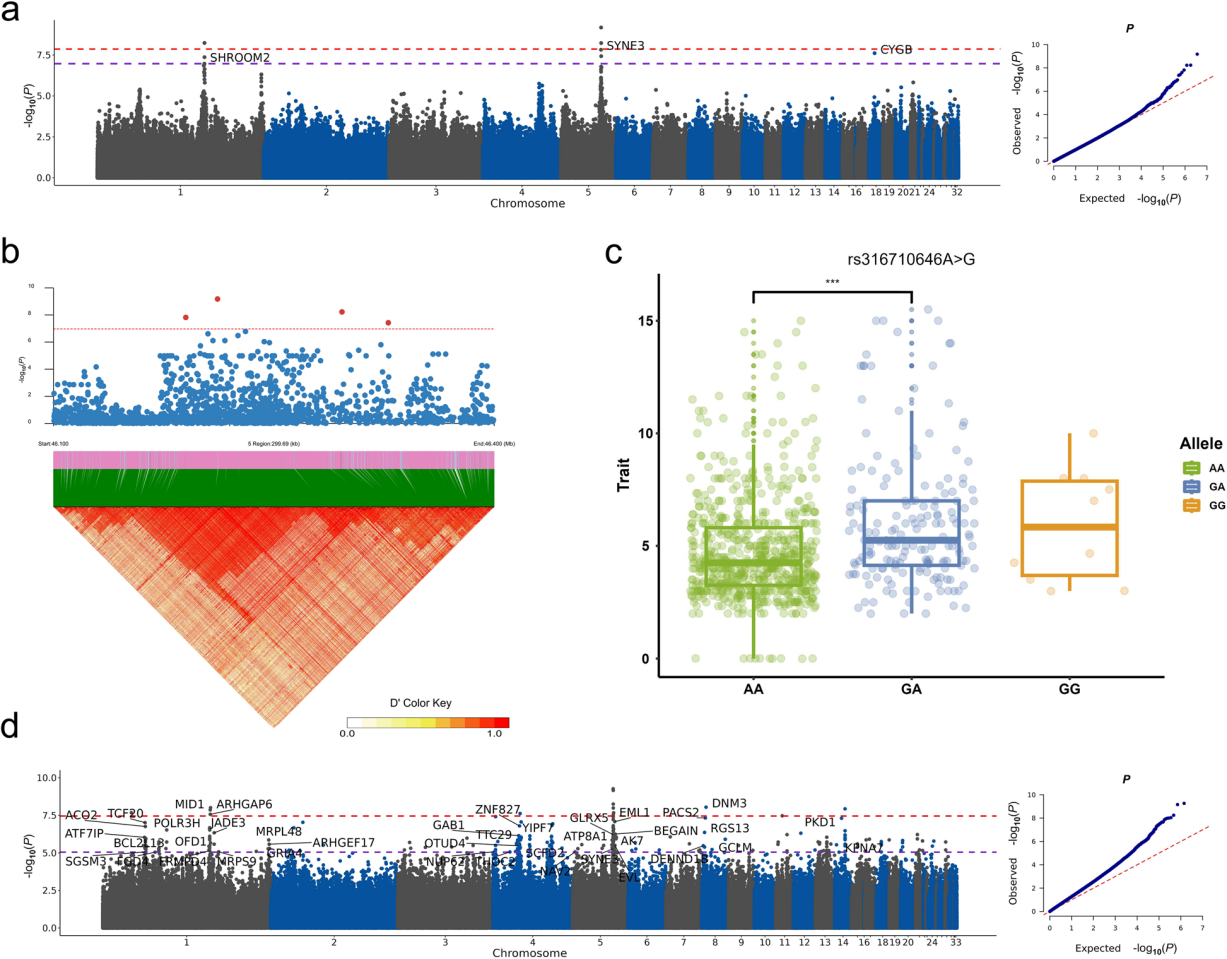
SNP-based and haplotype-based GWAS results for up-ECI trait. **a** Manhattan and Q-Q plots for SNP-based GWAS. Red dash line is the Bonferroni threshold and purple dash line is the 5% FDR threshold. Genes associated with significant SNPs were labeled. **b** Heatmap of LD pattern within the genomic region from 46.1Mb to 46.4 Mb on GGA5. The red dot represents significant SNP. It shows that the significant signals from the SNP-based GWAS in this region are in high LD. **c** Box plots of individuals carrying different genotypes of rs316710646 for up-ECI trait. ***P < 0.001. **d** Manhattan and Q-Q plots for haplotype-based GWAS for up-ECI trait

It is widely acknowledged the polygenic nature of complex traits. Across the three egg-laying stages, GWAS for most traits revealed no genome-wide significant signals (Supplementary Fig. S2). However, for the up-ECI (effective clutch intensity) trait, eight significant loci were identified on GGA1, GGA5, and GGA18 (Fig. 2a, Table 1). In the region spanning from 126.4 Mb to 126.5 Mb on GGA1, three SNPs, rs13935264 (*P* = 1.075e-07), rs13935293 (*P* = 5.841e-09) and rs739946136 (*P* = 4.321e-08), were annotated to the gene *SHROOM2*. In the region from 46.1 Mb to 46.4 Mb on GGA5, rs317474373 (*P* = 6.657e-10) and rs317620935 (*P* = 1.524e-08) were annotated to the gene *SYNE3*. The genomic region comprising the significant loci on GGA5 exhibited strong linkage disequilibrium (LD), suggesting high reliability of the associated signals (Fig. 2b). As an example, the three genotypes of rs316710646 showed ordinal changes in up-ECI trait, i.e., ${GG}_{up-ECI}> {GA}_{up-ECI}> {AA}_{up-ECI}$, suggesting the G allele to be beneficial for the trait (Fig. 2c). In addition, a significant SNP rs316748724 (*P* = 2.460e-08), located around 4.3 Mb on GGA18, was annotated to the gene *CYGB*. Some significant signals were detected for the sustained-TILI (total inter-laying interval) trait (Supplementary Fig. S3, Table 1). Three significant signals were located between 30.5 Mb and 30.7 Mb on GGA7, with the most significant SNP, GGA7_30617934 (*P* = 3.729e-09), annotated to the gene *R3HDM1*. Another significant SNP on GGA7, rs794029118 (*P* = 2.841e-08), was annotated to the gene *ZRANB3*. Moreover, rs1058884213 (*P* = 1.066e-08), around 38.2 Mb on GGA5, was annotated to the gene *LTBP2*.

**Table 1:** Summary statistics for significant SNPs in SNP-based GWAS

Chr	Pos	SNP	Beta	*P-*value	PVE	Trait	Gene
1	126,473,381	rs13935264	−1.321	1.075E-07	0.046	up-ECI	*SHROOM2*
1	126,499,631	rs13935293	−1.45	5.841E-09	0.061	up-ECI	*SHROOM2*
1	126,502,470	rs739946136	1.431	4.321E-08	0.053	up-ECI	*SHROOM2*
5	46,189,974	rs317620935	1.138	1.524E-08	0.038	up-ECI	*SYNE3*
5	46,211,585	rs317474373	−1.264	6.657E-10	0.038	up-ECI	*SYNE3*
5	46,296,173	rs316710646	1.176	5.923E-09	0.041	up-ECI	Intergenic
5	46,327,686	rs316819369	1.065	3.744E-08	0.029	up-ECI	Intergenic
18	4,344,887	rs316748724	2.292	2.46E-08	0.059	up-ECI	*CYGB*
5	38,150,466	rs1058884213	6.268	1.066E-08	0.036	sustained-TILI	*LTBP2*
7	30,547,659	rs794029118	6.020	2.841E-08	0.038	sustained-TILI	*ZRANB3*
7	30,570,758	rs315063973	5.801	1.614E-08	0.041	sustained-TILI	Intergenic
7	30,617,934	7_30617934	6.123	3.729E-09	0.040	sustained-TILI	*R3HDM1*

Only SNPs passed the genome-wide significant threshold were listed. SNP without an rsID means that it has not been previously reported in dbSNP. PVE, proportion of variance explained; up-ECI, effective clutch intensity trait during the up-stage; sustained-TILI, total inter-laying interval trait during the sustained-stage

Haplotype analysis may provide more power to detect associations as haplotypes can capture the combined effects of multiple linked loci [41]. Unlike conventionally additive SNP encoding, haplotypes are typically encoded as categorical variables. Genome-wide haplotype GWAS (HGWAS) was performed using a non-overlapping window with five-SNPs, for all 7,065,827 SNPs after phasing and pruning. HGWAS detected many association signals that had not shown significance in SNP-based GWAS. For the up-ECI trait, significant haplotype signals were predominantly occurred on GGA1, GGA4, GGA5, and GGA14 (Fig. 2d, Supplementary Table S2). New significant loci were found for all-TILI trait on GGA18 (Supplementary Fig. S4, Supplementary Table S3). HGWAS also revealed significant loci for all-WEV (weekly egg production variance) trait and up-MILI (maximum inter-laying interval) trait on GGA4, which were undetected in SNP-based GWAS (Supplementary Fig. S4, Supplementary Table S4 and S5).

As HGWAS only reports significant haplotype blocks, we employed separate general linear models (GLM) to estimate the effect size of each haplotype allele within these blocks, utilizing haplotype allele dosage coding. Beneficial haplotype alleles (BHA) and unbeneficial haplotype alleles (UHA) were classified based on their positive or negative effects on desirable egg-laying traits. For instance, for the up-ECI trait, 262 significant blocks were identified genome-wide, comprising 140 blocks containing BHA, 86 blocks containing UHA, and 10 blocks containing both. Lists of BHA and UHA for all traits with significant HGWAS signals are provided in Supplementary Table S6.

Both SNP-based GWAS and HGWAS test each locus independently. For multiple loci associated with the same trait, they can be incorporated in one model simultaneously to estimate the phenotypic variation jointly explained by them. For significant loci identified by SNP-based GWAS, a genomic relationship matrix (GRM) was constructed for each trait using SNPs from significant loci, and the corresponding proportion of variance explained (PVE) was computed by random effect. Similarly, haplotype alleles from the significant haplotype blocks identified by HGWAS were used to construct haplotype-based GRM and the corresponding PVE was calculated. For the up-ECI trait, the PVE from HGWAS was 38.7%, three-fold improvement over the 11.2% of PVE from SNP-based GWAS. For all traits with significant GWAS signals, lists of PVE estimates from significant loci identified by SNP-based and haplotype-based GWAS are provided in Table 2.

**Table 2:** Significant loci PVE and genome-wide PVE in GWAS

Trait	Significant genetic variance (${\sigma }_{{{\varvec{\mu}}}_{1}}^{2}$)	Insignificant genetic variance (${\sigma }_{{{\varvec{\mu}}}_{2}}^{2}$)	Significant loci PVE	Genome-wide PVE	Type
all-TILI	83.508	16.473	37.0%	44.3%	HGWAS
all-WEV	0.091	0.186	8.6%	26.3%	HGWAS
up-ECI	0.718	0.911	11.2%	25.5%	SNP-based GWAS
up-ECI	2.697	0.225	38.7%	42.0%	HGWAS
up-MILI	24.406	4.733	33.4%	39.9%	HGWAS
sustained-TILI	3.188	12.978	4.3%	21.7%	SNP-based GWAS

### Polygenic architecture for egg-laying traits

**Fig. 3:**
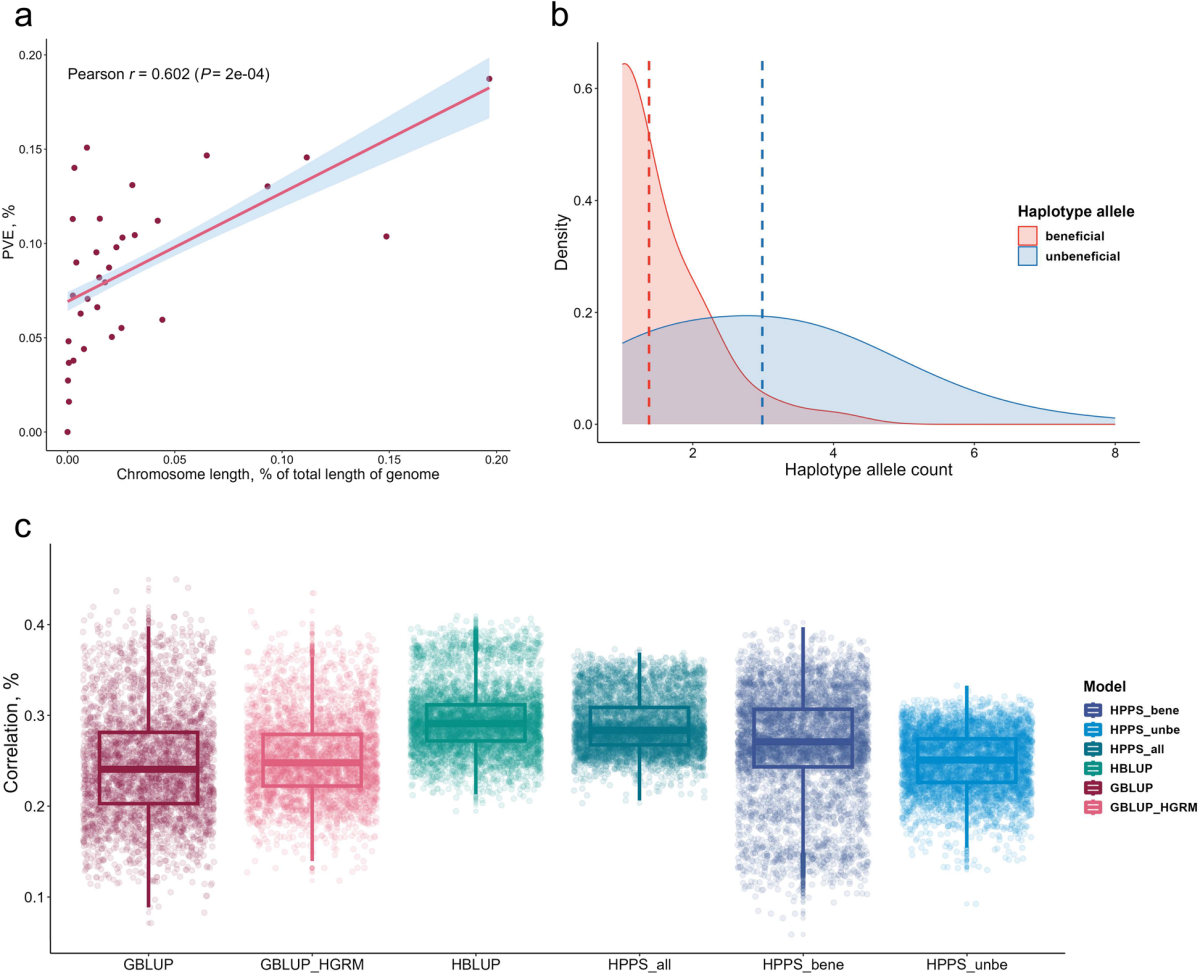
Polygenic architecture of egg-laying traits revealed by haplotype. **a** The PVE by each chromosome was scaled with its length. The red scatter indicates the PVE calculated for each chromosome, the red straight line is the regression line between chromosome length and PVE by each chromosome. The Pearson correlation coefficient of 0.6 indicates polygenic architecture for the up-ECI trait. **b** Density plot of BHA and UHA contained in significant haplotypic blocks. The *X*-axis is the haplotype allele count and the *Y*-axis is the distribution density. Dash line indicates the mean value of the corresponding distribution. **c** Prediction accuracy of HBLUP, three HPPSs, and two GBLUPs. Phenotype predictions based on significant haplotype blocks perform better than the widely used GBLUP models (particularly using SNP-based GRM). The HPPS_all method considering both BHA and UHA shows high performance. The HBLUP method shows best performance

A large number of genomic HGWAS signals indicated the polygenic nature of the egg-laying traits. Despite the significant signals reported, many loci with small effects remained unidentified under the GWAS cut-off. To account for the contributions of these loci, model with two random effects from both significant and insignificant loci was constructed. The model for haplotypes explained 42% of the phenotypic variance for the up-ECI trait, while the model for SNPs accounted for only 25.5%. For all traits with significant GWAS signals, lists of genome-wide PVE by SNPs and haplotypes are provided in Table 2. For polygenic traits, loci with small effects are distributed across chromosomes. Consequently, the PVE by each chromosome would correlate positively with its length. For the up-ECI trait, a strong positive correlation (*r* = 0.602) was indeed observed based on haplotypes, although some intermediate and small chromosomes exhibited disproportionately large PVEs relative to their length (Fig. 3a). We also examined the number of BHA and UHA within each significant haplotype block associated with egg-laying traits. It was found that significant haplotype blocks contained an average of 1.38 BHA and 2.99 UHA, highlighting the multi-allelic architecture of the egg-laying traits (Fig. 3b).

The polygenic architecture of the trait enables phenotype prediction using whole-genome data. For each HGWAS significant block, the effect size of each haplotype allele was firstly estimated as fixed effect using GLM. On this basis, the effect size for all significant blocks of diploid individuals were summed up and termed the haplotype-based polygenic prediction score (HPPS). Likewise, in a separate model, the haplotype allele effect was estimated as random effect, referred to as haplotype-based best linear unbiased prediction (HBLUP). The genomic best linear unbiased prediction (GBLUP) method, widely used in animal breeding, was also employed. The GBLUP approach, which does not require estimation of individual marker effects, was implemented using GRMs constructed from both genome-wide SNPs or haplotypes (Fig. 3c). The predictive performance was assessed by correlating the predicts with the original phenotype value. To reduce the risk of overfitting, evaluations were conducted against the constant validation set. Taking up-ECI trait as an example, based on 50 iterations of tenfold cross-validation, it was found that the traditional GBLUP method (R = 0.246) and GBLUP on the haplotype GRM (R = 0.253) did not perform very well. Notably, the HBLUP (R = 0.295) was slightly more accurate than HPPS_all (R = 0.289) which uses all haplotype alleles from the significant blocks. The HPPS using only BHA (R = 0.267) or UHA (R = 0.249) showed moderate performance. The amalgamation of BHA and UHA proved superior to segregating them, implying that including unbeneficial alleles aids in enhancing phenotype prediction.

### Tracking trait-associated haplotype alleles in chicken populations

**Fig. 4:**
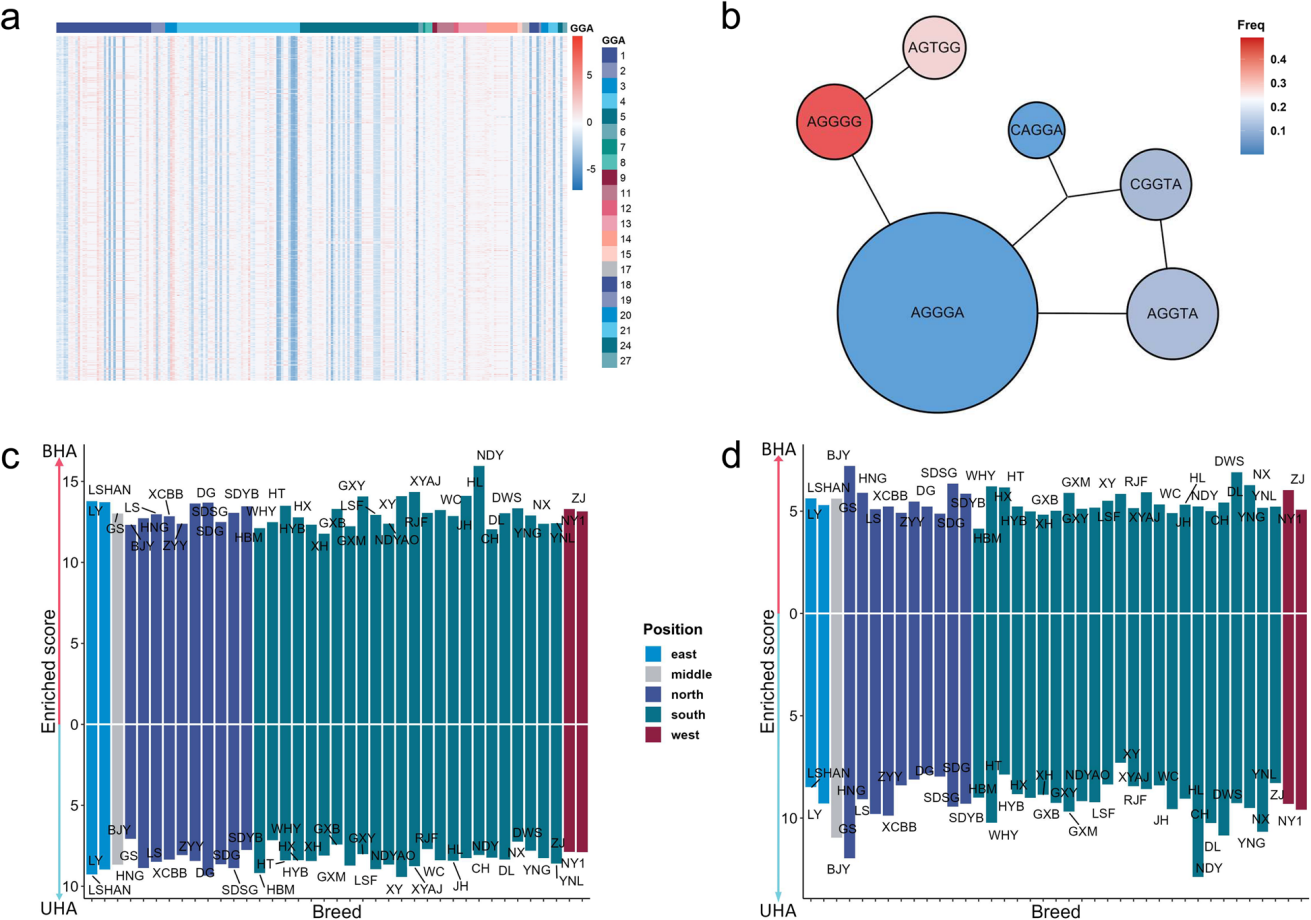
Analyses of significant haplotype alleles in up-ECI trait. **a** Heatmap of effect size of haplotype alleles within significant haplotype blocks. Each column represents a haplotype block, each row represents an individual. **b** Effect sizes and frequency of haplotype alleles in haplotype block No.173. The size of the circle indicates the effect size of haplotype alleles, and the color indicates its frequency in population. Lines connecting two haplotypes indicate one single base alternation between the two haplotype sequences. **c** The enriched score for haplotype alleles in 39 local chicken populations for up-ECI trait. Only BHA or UHA in HGWAS significant block were considered. The chicken populations are sorted by geographic locations. Blue indicates East China, grey indicates Central China, purple indicates North China, green indicates South China, and red indicates West China. **d** The enriched score for haplotype alleles in 39 local chicken populations for up-MILI trait. Only BHA or UHA from HGWAS significant block were considered

As shown in Fig. 4a and Supplementary Fig. S5, numerous haplotype alleles were effective on up-ECI trait across the genome. By comparing sequences between haplotype alleles within a haplotype block, we found mutations on some haplotype alleles significantly alter the degree of associations with trait. For example, in haplotype block No.173 (genomic position GGA5: 46,764,467–46,765,093 bp) that showed significant association with the up-ECI trait, a G > A mutation changed the haplotype allele “AGGGG” to “AGGGA”. Despite the frequency of the derived haplotype allele “AGGGA” was low (f = 0.001), the effect size increased from 0.337 (“AGGGG”) to 5.788 (“AGGGA”) (Fig. 4b), suggesting that this BHA may appear recently. In contrast, a BHA “GACAC” at low frequency (f = 0.037) but a high effect size of 2.0 within haplotype block No. 247 (genomic location GGA18: 4,206,126–4,206,301 bp), corresponding to gene *MGAT5B,* had limited identity with the dominant haplotype allele “GGAGT” (f = 0.856) with an effect size of only 0.243 (Supplementary Fig. S6). Surprisingly, the BHA “GACAC” had the highest frequency (45%) in the Red Jungle Fowl (RJF) and showed a trend of decreasing frequency from the South to the North in domestic chickens (Supplementary Fig. S7). A functional survey revealed that *MGAT5B* promotes egg production by up-regulating the expression of egg-laying related genes *AKR1D1* and *EDA2R* [42]. However, *MGAT5B* has also been implicated in neurological disorders, where its deficiency led to decreased astrocyte activation and enhanced oligodendrocyte maturation [43]. We suspected that *MGAT5B* has been subject to negative selection in local chicken populations, resulting in a trade-off that sacrifices some egg yield to potentially reduce the incidence of neurological disorders.

We further examined the frequencies of BHA and UHA identified in all egg-laying traits of the Gushi chicken among 39 Chinese local chicken populations (Supplementary Table S7). The Ningdu Yellow chicken (NDY) was found to have the highest sharing rate of BHA (${E}_{\text{NDY}}^{\text{BHA}}$= 16.015) for the up-ECI trait and the highest sharing rate of UHA (${E}_{\text{NDY}}^{\text{UHA}}$= 12.925) for the up-MILI trait (Fig. 4c and d). This suggests substantial genetic exchange between the NDY and the Gushi chicken, which is not surprising given that two breeds are distributed in close geographic proximity. Interestingly, the Beijing-You chicken (BJY) in northern China exhibited high degree of shared haplotype alleles (${E}_{\text{BJY}}^{\text{BHA}}$= 1.947) associated with increased phenotypic variance in the all-WEV HGWAS. In contrast, the two southern chicken populations (Huaibei Partridge chicken (HBP) and Dulong chicken (DL)) showed a high degree of shared haplotype alleles (${E}_{\text{HBP}}^{\text{UHA}}$= 1.513, ${E}_{\text{DL}}^{\text{UHA}}$= 1.438) that decrease phenotypic variance (Supplementary Fig. S8). The lower egg production variance of southern Chinese chickens may be attributed to the uniform sunshine and temperature in southern China, which creates suitable conditions for year-round egg production. Conversely, northern Chinese chickens exhibited relatively high egg production variance, potentially reflecting adaptation to greater variability of the environment in the North.

### Polygenic selection on egg production efficiency

Domesticated chickens are subjected to both natural and artificial selection. Given the high polygenicity of egg-laying trait, the response to selection would differ from that of traits influenced by a few high-impact loci. As expected, only few significant selective signals on GGA6 were detected by iHS approach (Supplementary Fig. S9). The haplotype-based H12 statistic yielded similar results and showed no signal of purifying selection (Supplementary Fig. S10). However, Tajima's D scores ranged between 2 and 4 across most genomic regions, indicating a significant deviation from the neutral expectations (${P}_{t.test(\text{Tajima}^{\prime}\text{s D}=0)} < 0.01$) (Supplementary Fig. S11).

**Fig. 5:**
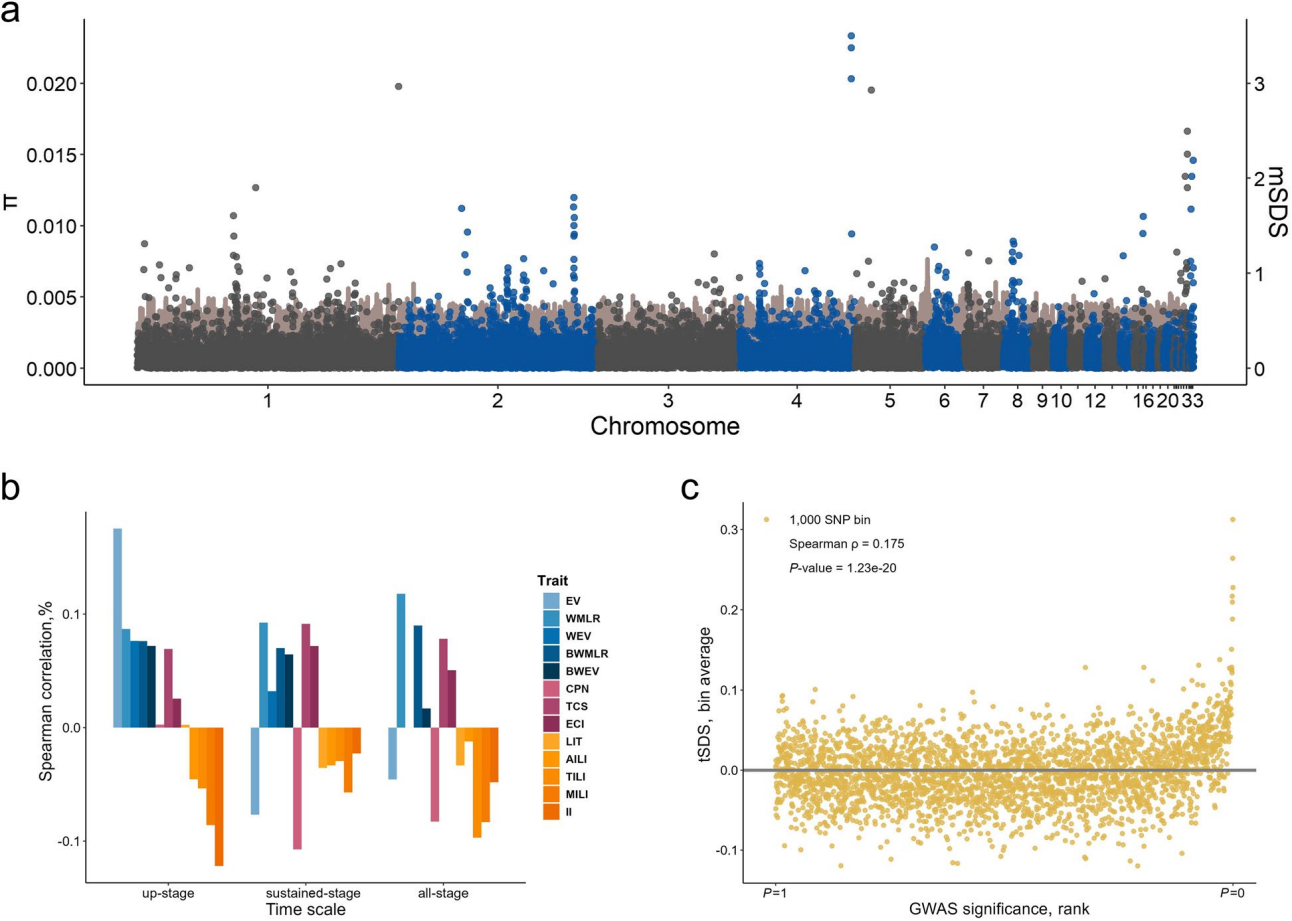
Polygenic selection in chicken population. **a** The rSDS Z-scores were converted into *P*-values based on 1% AF bin to characterize the degree of selection. The mSDS was the mean value of the Z-scores within 50 kb fixed window. Blue and grey scatters indicate the mean SDS scores in each window, and brown lines indicate the average π values. Multiple chromosomes had high mSDS peaks, suggesting the presence of a relatively strong selected locus. **b** Spearman correlation coefficients between GWAS *P*-value ranks and tSDS for all traits during three stages. Stability and clutch related traits showed heterogeneity across laying stages, and interval-related traits were subjected to negative selection in all three stages. Production variance traits were subjected to positive selection during the up-stage, in contrast to the other stages. **c** Scatterplot of tSDS against GWAS *P*-values for up-EV trait. SNPs with high significant GWAS *P*-values also have high tSDS

To better quantify selection for complex traits, the singleton density score (SDS) was estimated for all SNPs in individual genomic windows of 50 kb. The raw SDS (rSDS) results showed that the Gushi chicken was subjected to polygenic selection over the past ~100 years (Fig. 5a). In the high mSDS region on GGA2, we observed relatively low π and high iHS values. As iHS detects selection signals that occurred approximately 1,000 years ago while SDS captures recent selection signals within the last 100 to 200 generations, the pattern suggests that this region has been under selection over last 100 years. Conversely, in the high mSDS region on GGA27, both iHS and π values were low, indicating that the selection on this region took place only recently (Supplementary Fig. S12).

We polarized the sign of the rSDS according to the GWAS result for each trait, so a positive trait-SDS (tSDS) indicates an increase in the frequency of allele favoring the trait. For polygenic traits, SNPs that do not reach genome-wide significance in GWAS also contribute to phenotypic variations. Therefore, in the tSDS analysis, we considered all SNPs rather than only significant SNPs in GWAS. For each trait, the Spearman correlation between −log_10_(*P*) from SNP-based GWAS and tSDS values was computed (Fig. 5b, Supplementary Table S8). Theoretically, if a trait is favored by polygenic selection, a positive coefficient is expected; otherwise, the correlation would be negative. We found that the Gushi chicken was subjected to different polygenic selections at different stages: EV trait experienced positive selection during the up-stage (Fig. 5c), while it was selected against during the sustained- and all-stages (Fig. 5b). We hypothesized that during the up-stage, artificial selection favors higher egg production variance to facilitate accelerated productivity growth, whereas in the sustained-stage, lower egg production variance is favored to sustain prolonged egg production at a satisfactory level. Moreover, the frequent alternations between clutches and intervals may interrupt egg-laying continuity, leading to the observation that the CPN (clutch period number) trait was selected against, whereas the ECI trait was favored by selection during the sustained-stage (Fig. 5b). These pieces of evidence collectively indicate refined polygenic selection for enhanced egg production efficiency.

Using a similar approach to correlate HGWAS summary statistics with SDS, we found that SNPs corresponding to BHA had a positive mSDS, whereas SNPs corresponding to UHA had a negative mSDS. We found that polygenic selection was even more pronounced at the haplotype level: the average tSDS for the up-ECI HGWAS significant block was 0.160, much higher than that for SNPs (0.026). This suggests that the polygenic selection on egg production efficiency is better captured using haplotypes.

### Dual role of *CNNM2* on egg production variance to optimize egg production efficiency

**Fig. 6:**
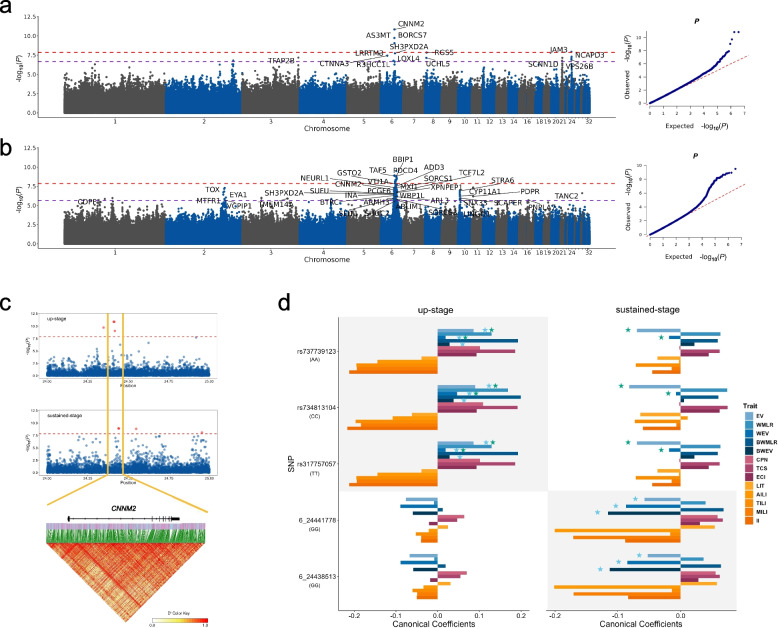
Multi-trait CCA-based GWAS results during the up-stage and sustained-stage. **a** Manhattan and Q-Q plots for CCA-based GWAS in the up-stage. Red dash line is the Bonferroni threshold and purple dash line is the 5% FDR threshold. **b** Manhattan and Q-Q plots for CCA-based GWAS during the sustained-stage. A large number of significant signals were identified on GGA6, and some of the significant peaks were also identified at GGA2 and GGA10. **c** Profile of significant signals in *CNNM2* gene. Association signals during the up-stage and sustained-stage did not overlap but were in strong LD. **d** Canonical coefficients between SNP genotypes and all traits during the up-stage and sustained-stage. Shaded areas highlight SNPs within the *CNNM2* gene that showed statistical significance in CCA-based GWAS. Egg production variance-related traits showed significant positive correlations with genotypes of three SNPs during the up-stage, while exhibiting significant negative correlations with genotypes of two other SNPs during the sustained-stage. Green stars denote dual role of genotypes on egg production stability during the two stages, whereas blue stars denote two SNP sets within the *CNNM2* gene that showed dual role during the two stages

Since loci that promote multiple egg-laying traits related to egg production efficiency were selectively favored, this suggests that certain genes or loci may have pleiotropic effects on egg production efficiency. Canonical correlation analysis (CCA)-based GWAS was employed to test the correlation between each SNP and multiple traits simultaneously in the up-stage and sustained-stage. Upon a 5% false discovery rate (FDR) correction, 22 significant SNPs were identified during the up-stage, of which 7 were located between 23.2 Mb and 25.0 Mb on GGA6 (Fig. 6a, Supplementary Table S9). A total of 166 significant SNPs were detected during the sustained-stage, among them 133 were located between 23.3 Mb and 28.9 Mb on GGA6 (Fig. 6b, Supplementary Table S10). The significant signals on GGA6 in both the up-stage and sustained-stage were mapped to the same gene *CNNM2*, which spans approximately 100 kb and harbors three associated SNPs in its intron 1 during the up-stage and two associated SNPs in its introns 2 and 4 during the sustained-stage. Although these five significant SNPs did not physically overlap (being about 20 kb apart), they were in strong LD (Fig. 6c).

For each of the five SNPs, the canonical coefficients between the SNP genotypes and each trait were calculated to reflect the relative contribution of the SNP effects on the individual trait (Fig. 6d). The three up-stage associated SNPs in its intron 1 were positively correlated with production variance (EV, WEV, BWEV) during the up-stage, while EV and WEV show negative correlation with the same genotypes during the sustained-stage. As an example, the CC genotype of rs734813104 increases the EV during the up-stage, but decreases it during the sustained-stage, indicating dual role of this genotype during the two stages. On the other hand, the two sustained-stage-associated SNPs in introns 2 and 4 are negatively correlated with production variance, contributing to stability during the sustained-stage (Fig. 6d). Considering the strength and direction of effects of the three up-stage associated SNPs, the five SNPs together constitute a dual role of the *CNNM2* gene on egg production variance during the two stages, which aligns with patten of polygenic selection observed.

To further elucidate the stage-dependent genetic mechanisms underlying egg production, we employed an alternative approach by constructing linear models to pinpoint specific interacting genotype with laying stage on EV trait. The three up-stage associated SNPs identified through CCA-based GWAS showed significant genotype-by-stage interactions (*P* = 0.008, 0.008, 0.005). Post-hoc analysis revealed that during the up-stage, the CC genotype of rs734813104 was significantly associated with increased EV (effect size = 0.94, *P* = 0.01), while the TT genotype was significantly associated with decreased EV (effect size = −1.59, *P* = 0.03), supporting the results from the canonical coefficients.

Altogether, *CNNM2* exhibits a dual role at both the SNP and gene levels on egg production variance during the up-stage and sustained-stage of the egg-laying cycle to optimize egg production efficiency.

### *CNNM2* is functionally important in egg-laying

**Fig. 7:**
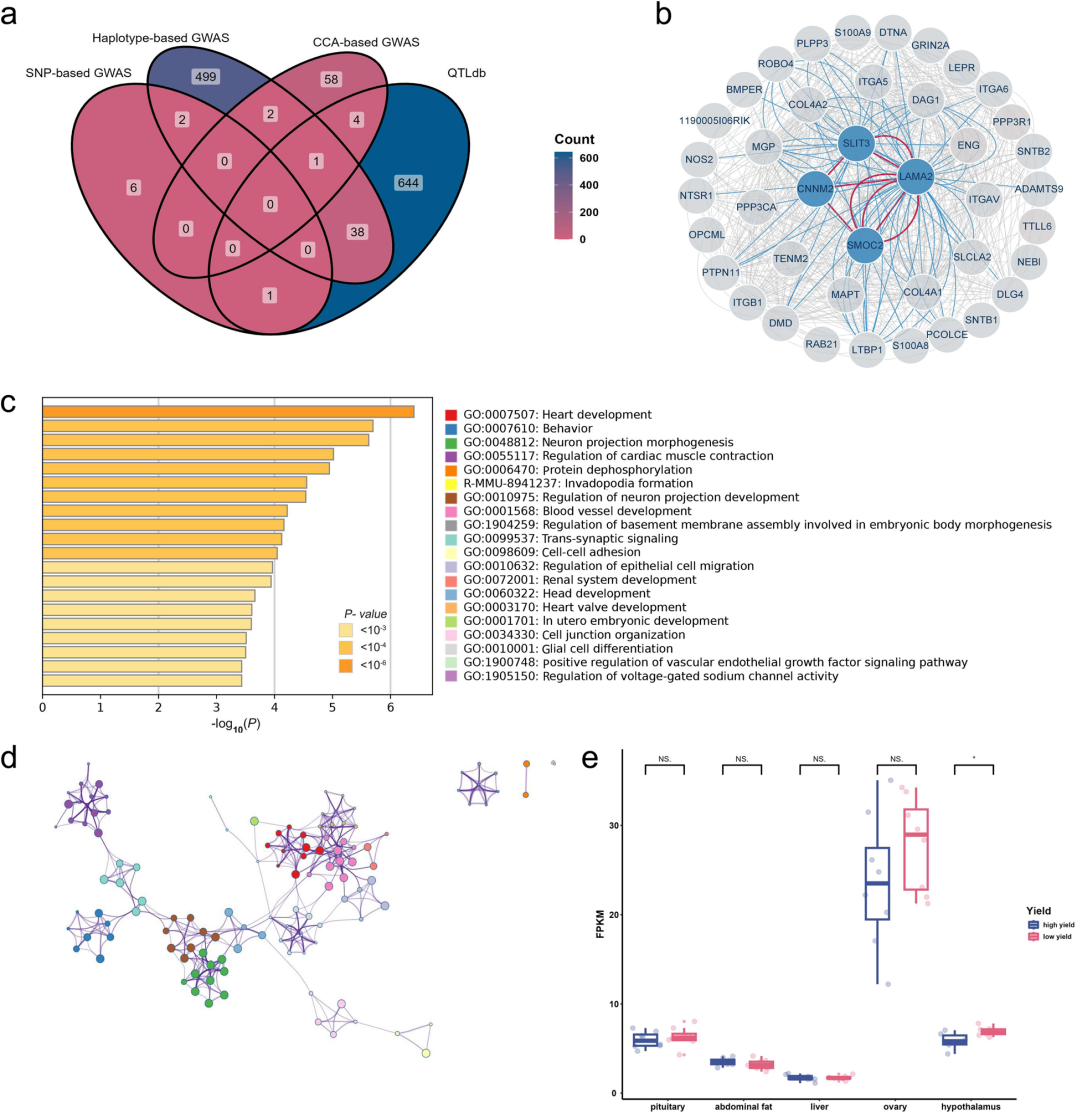
Functional analyses for GWAS candidate genes. **a** Venn diagram for reproduction-related genes in chicken QTLdb and candiated genes from three GWAS methods. **b** Integrative biology network built from *CNNM2* and top 20 prioritized genes. Gene is represented as network nodes, blue lines indicate interacting between hub genes and other genes, red lines indicate interacting between hub genes, gray lines indicate interacting between other genes. **c** Bar chart of GO enrichment for all GWAS candidate genes. The color of bar indicates the *P*-value of enrichment terms, the *X*-axis is the significance of the enrichment, and the *Y*-axis is the enrichment terms. The most enriched terms include heart development, neurodevelopment, myocardial contraction, etc., revealing the major genetic mechanisms underlying egg-laying traits. **d** GO-enriched network constructed by similarities of enriched terms. The nodes represent various enrichment terms in **c**, with corresponding colors. The edges indicate similarities among terms greater than 0.3. **e** *CNNM2* expression in high-yielding and low-yielding chickens. *CNNM2* showed significant differences in the hypothalamus between high-yielding and low-yielding chickens

In this study, a large number of candidate genes were identified through different mapping approaches. Haplotype-based GWAS identified the highest number of candidate genes (542), followed by CCA-based GWAS (65), and SNP-based GWAS identified the fewest (9). Two candidate genes, *LTBP2* and *SYNE3*, were found in both haplotype-based and SNP-based GWAS, and three candidate genes, *EYA1*, *BTRC*, and *PNPLA7*, were found in both haplotype-based and CCA-based GWAS (Fig. 7a). The candidate genes reported in this study partially overlapped with the reproduction-related genes in the chicken QTLdb (Fig. 7a). Moreover, the genes identified through SNP-based and haplotype-based GWAS showed substantial overlap with those from our previous study, despite the derived egg-laying traits not being exactly the same (Supplementary Fig. S13). Notably, when applying the same significance threshold as in our earlier work, we successfully replicated significant associations with genes such as *TFPI2, CAMK2D*, *OSTN* and *APOA4* (Supplementary Table S11). GO enrichment analysis of all candidate genes using Metascape (Fig. 7c and d) revealed that the enriched terms including heart development, vascular development, neuromodulation, embryonic development, and cell adhesion. Notably, these functional categories aligned with the known roles of the HPO axis [44].

The candidate genes were further prioritized to determine their biological relevance, in which known genes associated with human reproduction traits in the GWAS catalog were used as the reference set. A total of 419 candidate genes were successfully prioritized. The top 20 prioritized genes (Supplementary Table S12), along with *CNNM2*, were then selected to construct an integrative biology network (Fig. 7b). Notably, *CNNM2* occupies a central position in this network by interacting with other genes through three hub genes: *SLIT3*, *LAMA2*, and *SMOC2*. We checked the gene expression of *CNNM2* across multiple tissues. It is believed that mutations located within introns can affect splicing and gene expression. Since the Illumina sequencing platform we used is not well-suited for accurately detecting alternative splicing, we tried to investigate the correlation between the genotypes of five associated intron SNPs, as well as SNPs in high LD with them, and the expression of *CNNM2*. Unfortunately, we were unable to find any significant correlation. We did find that *CNNM2* was highly expressed in the ovary (Fig. 7e). By comparing the expression of *CNNM2* between high-yield and low-yield chickens, we observed significant lower expression of *CNNM2* in hypothalamus of high-yield chickens. This finding confirms that *CNNM2* plays a role in egg-laying in chickens (Fig. 7e).

## Discussion

GWAS has become a standard approach for genetic mapping. In this study, many candidate genes were identified through GWAS on up-ECI and sustained-TILI traits. The annotated genes function directly or indirectly to the egg production. Among them, *SHROOM2* is highly expressed in the endothelium of the developing vasculature, playing a crucial role in the initial formation and subsequent remodeling of vascular network [45, 46]. *SYNE3* on the other hand, is essential for perinuclear cytoskeletal organization and the attachment of centrosome to nuclear envelope [47]. It forms spermiogenesis-specific LINC complexes with Sun1η [48], suggesting its involvement in the process of sperm development. *CYGB*, a cytoglobin, may be involved in collagen synthesis or in the function of O^2^-consuming enzymes, facilitating O^2^ diffusion to respiratory chain [49, 50]. *R3HDM1* affected the formation of neural networks in brain, modulating the growth and branching of dendrites in mouse normal neurons [51]. Additionally, *R3HDM1* has been identified as one of the up-regulated genes in the pregnant endometrium, suggesting its involvement in reproductive regulation [52]. Some associated genes seem to have no direct relation to egg-laying, this may be due to the insufficiency of our current understanding of reproductive processes. For example, *ZRANB3* is typically involved in maintaining genomic stability during DNA replication [53]. *LTBP2* plays a positive role in lung elastinogenesis [54]. In summary, these genes correspond to various functions, suggesting complex molecular mechanisms underlying egg production.

While GWAS has been highly successful, SNP-based GWAS has known limitations in fully uncovering the genetic basis of complex traits [55]. Haplotype-based GWAS aggregates the effects of multiple linked loci, potentially combining additive, dominant, and short-range epistatic effects together. Studies have shown that haplotype-based GWAS is superior to SNP-based GWAS in terms of statistical power, allelic effect estimation, and avoiding false-positives [56–58]. In our study, haplotype-based GWAS detected more association signals that were undetected in SNP-based GWAS for egg-laying traits. In addition, for both aspects of total number of loci influencing the trait and the distribution of their effect sizes, the haplotype-based analysis well demonstrated the polygenicity of egg-laying traits.

Although selection signals are often considered to be significant changes in allele frequencies at high-impact loci, for polygenic traits most alleles are of small effects, phenotypic improvement can be achieved by the accumulation of the polygenic effects [59]. Without the need for fixation or dramatic changes in allele frequencies, populations can also shift allele frequencies at multiple loci to achieve optimal fitness [60, 61]. From this viewpoint, polygenic traits can respond rapidly to selection based on existing standing variations [62, 63]. The chickens were subjected to polygenetic selection of increasing egg production variance during the up-stage, which covers the period from the start of egg-laying to the peak production. Larger variance implies greater potential and faster increase in productivity, that is, individuals with large egg production variance are favored in the early stages of egg-laying, possibly due to selection for achieving rapid productive output. Production stability is more important during the sustained-stage, so egg production variance is selected against during this stage. Stable long-term egg production aligns with the breeding goal of modern chickens [64], so individuals with smaller egg production variance are favored to maintain a longer egg-laying cycle and higher egg production. The five associated SNPs in the *CNNM2* gene exhibited consistent characteristics. Three up-stage associated SNPs in *CNNM2* promote production variance, facilitating more rapid gains in production capacity from the start of egg-laying in chicken population. Conversely, two sustained-stage associated SNPs in *CNNM2* decrease variance during this period, resulting in a smooth decline in the laying curve and maintaining higher stability and overall efficiency in egg production.

From a gene functional perspective, *CNNM2* encodes a protein that plays an important role in magnesium homeostasis. It is widely recognized that magnesium plays a crucial role in neural conduction and neuronal signaling. Mutations in *CNNM2* are associated with hypomagnesemia, seizures, and impaired intellectual development [65]. In addition to genetic mechanisms, we explored the relationship between gene expression and egg-laying performance. As the expression of *CNNM2* in hypothalamus is significantly lower in high-yield chickens, we proposed two hypotheses: (1) decreased expression of *CNNM2* may reduce sensitivity to external stimuli by impairing cognitive function [66–71], or (2) reduced *CNNM2* expression may redirect energy allocation from growth to reproduction, thereby enhancing egg production [72, 73].

We identified several genes that preferentially interact with *CNNM2*. Among them, *LEPR* encodes the receptor of leptin. While leptin is primarily known as an appetite regulator, it contributes to the initiation and maintenance of reproductive activity by promoting gonadotropin secretion through the hypothalamus [74–77]. It has been reported that leptin inhibits follicular development by directly antagonizing ovarian estradiol and progesterone secretion stimulated by follicle-stimulating hormone (FSH) or insulin-like growth factor I (IGF-I) [78–80]. *ITGB1* plays a critical role in the migration of primordial germ cells to embryonic gonads in mice [81]. In spermatogenesis, both *ITGA6* and *ITGB1* serve as surface markers for mouse lymphocytes and spermatogonia [82]. *SLIT3* may play an inhibitory role in the proliferation, differentiation, and follicular selection of granulosa cells in the prehierarchical follicles in the ovary [83]. *LAMA2* exists in the neurons of the brain, regulates synaptic function and plasticity in the central nervous system [84]. *SMOC2* encodes a secreted protein that exhibits a broad tissue distribution in embryonic and adult mice [85]. It has been reported that *SMOC2* participates in the regulation of cell proliferation and angiogenesis [86]. Considering the findings of this study, we not only confirmed the polygenicity of egg-laying traits but also identified several significant GWAS signals associated with egg production efficiency and stability. We found that the combination of the two contribute a substantial proportion to the phenotypic variation. Our results agreed with the refined omnigenic model of egg production, which emphasizes the importance of core genes with large effects as well as the cumulative small effects across peripheral genes [87, 88].

## Conclusions

This study makes three key contributions to advancing our understanding the genetic mechanisms underlying egg production. Firstly, we demonstrated the advantages of haplotypes in genetic mapping, elucidating polygenic architecture, tracing ancestral origins, detecting polygenic selection and predicting phenotypes. Secondly, by utilizing traits generated to characterize egg production efficiency and stability, we identify key genes that affect these traits. Thirdly, by investigating polygenic selection signals of complex traits and applying multiple association analyses, we uncover the genetic basis of egg production efficiency. Our results provide valuable insights into enhancing egg production in chickens and position chickens as a model for studying the genetics of reproductive efficiency in other species.

## Supplementary Information

Additional file 1: Supplementary Table S1 Definition and descriptive statistics of derived traits. Supplementary Table S2 Haplotype-based GWAS summary statistics for up-ECI. Supplementary Table S3 Haplotype-based GWAS summary statistics for all-TILI. Supplementary Table S4 Haplotype-based GWAS summary statistics for all-WEV. Supplementary Table S5 Haplotype-based GWAS summary statistics for up-MILI. Supplementary Table S6 Distribution of BHA and UHA within significant blocks of haplotype-based GWAS. Supplementary Table S7 Information of 39 Chinese local chicken populations. Supplementary Table S8 Correlation between the rank of P-value from SNP-based GWAS and tSDS values. Supplementary Table S9 CCA-based multi-trait GWAS summary statistics for up-stage. Supplementary Table S10 CCA-based multi-trait GWAS summary statistics for sustained-stage. Supplementary Table S11 Candidate genes using significance thresholdin our previous study. Supplementary Table S12 Gene prioritization of candidate genes reported in SNP-based GWAS and haplotype-based GWAS

Additional file 2: Supplementary Fig. S1 Trait distribution and correlations of 39 traits. Supplementary Fig. S2 Manhattan plots of SNP-based GWAS for the all 39trait. The red dash line is the Bonferroni threshold and purple dash line is the 5% FDR threshold. The SNP-based GWAS of most derived trait had few significant signals. Supplementary Fig. S3 Manhattan and Q-Q plots of SNP-based GWAS for the sustained-TILI trait. Supplementary Fig. S4 Genome-wide haplotype-based GWAS results for other traits with significant signals. Supplementary Fig. S5 Density plot of effect size of haplotype alleles in significant haplotype blocks for the up-ECI trait. Supplementary Fig. S6 Effect size and frequency of haplotype alleles in haplotype block NO.247. Supplementary Fig. S7 Frequency of beneficial haplotype allele for the up-ECI trait in Haplotype NO.247 in 39 Chinese local chicken populations. Supplementary Fig. S8 The enriched score for haplotype alleles in 39 local chicken populations for the all-WEV trait. Supplementary Fig. S9 Genome-wide iHS signals for the chicken population. Supplementary Fig. S10 Genome-wide H12 signals for the chicken population. Supplementary Fig. S11 Genome-wide Tajima's D signal of the chicken population. Supplementary Fig. S12 SDS, π, and iHS values for 50 K significant blocks on high mSDS region. Supplementary Fig. S13 The overlap of SNP-based GWAS and haplotype-based GWAS between this study and our previous study. Supplementary Fig. S14 Egg-laying data before and after random forest imputing. Supplementary Fig. S15 PCA projections of 888 chickens

## Data Availability

The resequenced raw data from 888 Gushi hens were deposited in the Genome Sequence Archive (GSA) with accession number PRJCA021392. The RNA-seq raw data of 5 tissue types were deposited in NCBI Sequence Read Archive with accession number PRJNA893445 and PRJNA953784. All other data supporting the findings of this study are available within the article and its Supplementary Information files. Source data are provided with this paper.

## Abbreviations

AILI: Average inter-laying interval
BHA: Beneficial haplotype alleles
BJY: Beijing-You chicken
BLUP: Best linear unbiased prediction
BWEV: Bi-weekly egg production variance
BWMLR: Bi-weekly maximum laying rate
CCA: Canonical correlation analysis
CPN: Clutch period number
CV: Cross-validation
DAF: Derived allele frequency
DL: Dulong chicken
ECI: Effective clutch intensity
EV: Egg production variance
FDR: False discovery rate
GLM: General linear models
GRM: Genomic relationship matrix
GWAS: Genome-wide association study
HBP: Huaibei Partridge chicken
HGWAS: Genome-wide haplotype GWAS
HPO: Hypothalamic-pituitary-ovary
HPPS: Haplotype-based polygenic prediction score
iHS: Integrated haplotype score
II: Interclutch interval
LIT: Laying interval time
MILI: Maximum inter-laying interval
NDY: Ningdu Yellow chicken
PCs: Principal components
PVE: Proportion of variance explained
QTL: Quantitative trait loci
RJF: Red Jungle Fowl
SDS: Singleton density score
TCS: Total clutch size
TILI: Total inter-laying interval
UHA: Unbeneficial haplotype alleles
WEV: Weekly egg production variance
WMLR: Weekly maximum laying rate
