# Supplementary material for: Genome-wide analyses reveal intricate genetic mechanisms underlying egg production efficiency in chickens
Source: J Anim Sci Biotechnol. 2025 Aug 11;16:114. doi: 10.1186/s40104-025-01245-2 (PMC12337387; doi:10.1186/s40104-025-01245-2)
Supplement: Supplementary file 2 — Additional file 2: Supplementary Fig. S1 Trait distribution and correlations of 39 traits. Supplementary Fig. S2 Manhattan plots of SNP-based GWAS for the all 39trait. The red dash line is the Bonferroni threshold and purple dash line is the 5% FDR threshold. The SNP-based GWAS of most derived trait had few significant signals. Supplementary Fig. S3 Manhattan and Q-Q plots of SNP-based GWAS for the sustained-TILI trait. Supplementary Fig. S4 Genome-wide haplotype-based GWAS results for other traits with significant signals. Supplementary Fig. S5 Density plot of effect size of haplotype alleles in significant haplotype blocks for the up-ECI trait. Supplementary Fig. S6 Effect size and frequency of haplotype alleles in haplotype block NO.247. Supplementary Fig. S7 Frequency of beneficial haplotype allele for the up-ECI trait in Haplotype NO.247 in 39 Chinese local chicken populations. Supplementary Fig. S8 The enriched score for haplotype alleles in 39 local chicken populations for the all-WEV trait. Supplementary Fig. S9 Genome-wide iHS signals for the chicken population. Supplementary Fig. S10 Genome-wide H12 signals for the chicken population. Supplementary Fig. S11 Genome-wide Tajima's D signal of the chicken population. Supplementary Fig. S12 SDS, π, and iHS values for 50 K significant blocks on high mSDS region. Supplementary Fig. S13 The overlap of SNP-based GWAS and haplotype-based GWAS between this study and our previous study. Supplementary Fig. S14 Egg-laying data before and after random forest imputing. Supplementary Fig. S15 PCA projections of 888 chickens [file 40104_2025_1245_MOESM2_ESM.pdf]

Supplementary Figures

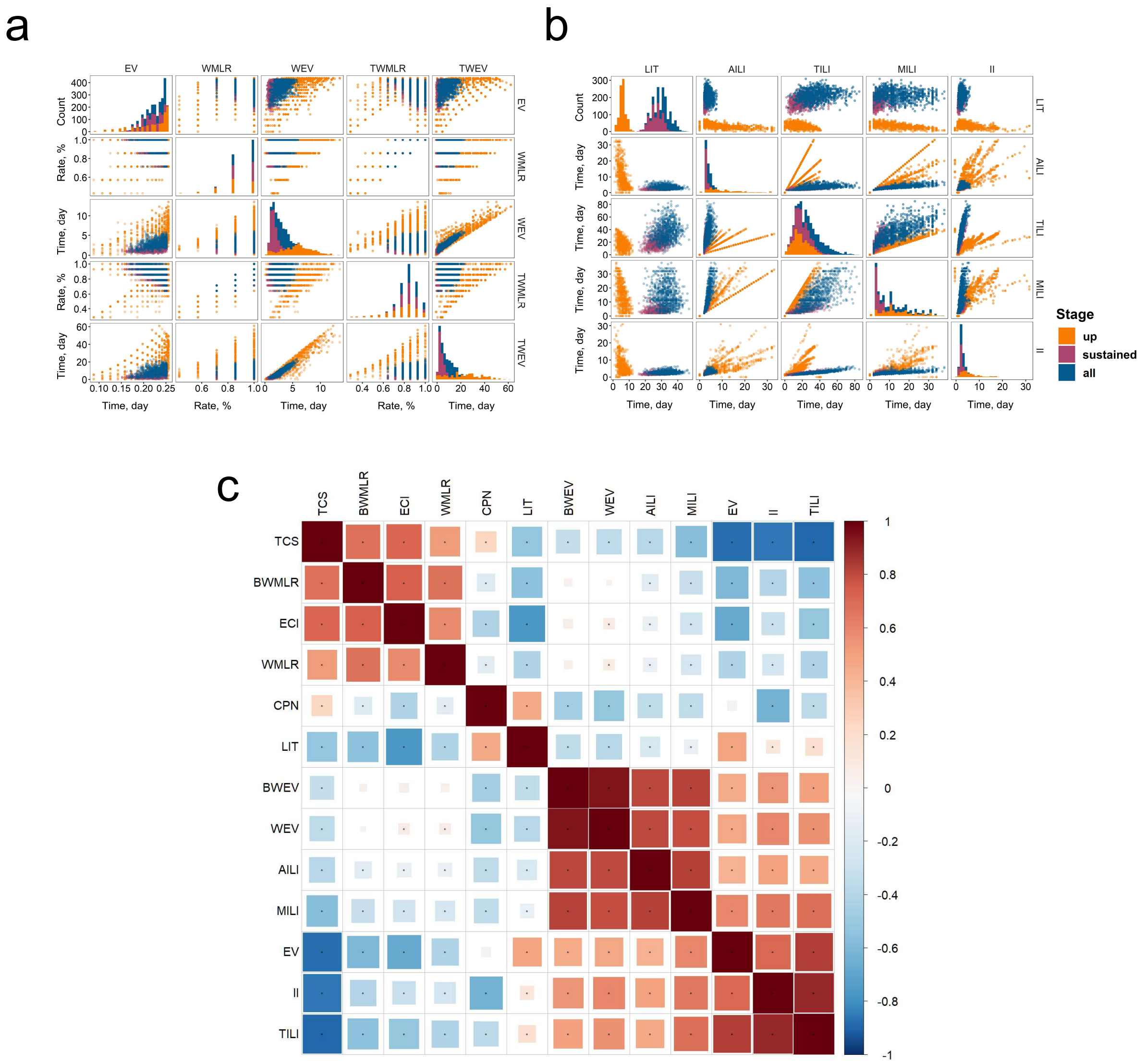

Supplementary Fig. S1. Trait distribution and correlations of 39 traits. a) Trait distribution of three clutch traits at three stages. Diagonal lines indicate single trait distributions, and upper and lower indicate scatter plots between two traits. The three colors indicate different stages. b) Trait distribution of five interval traits at three stages. c) Heatmap of Pearson correlation coefficients between 13 derived traits for the all-stage (21-43 weeks of egg production). Red color denoting coefficient > 0, blue denoting coefficient < 0, and * denoting the significant correlations between traits.

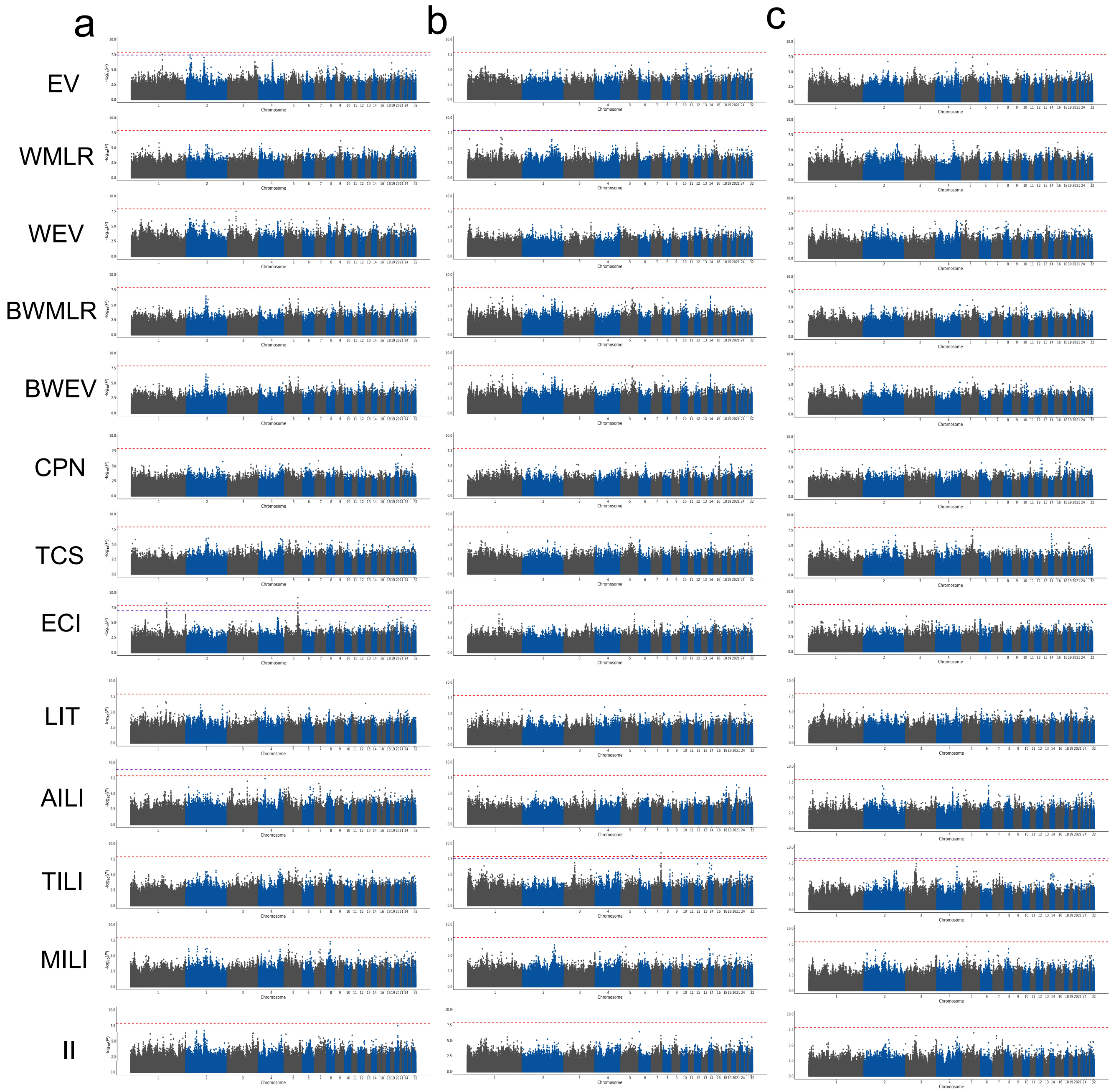

Supplementary Fig. S2. Manhattan plots of SNP-based GWAS for the all 39trait. The red dash line is the Bonferroni threshold and purple dash line is the 5% FDR threshold. The SNP-based GWAS of most derived trait had few significant signals.

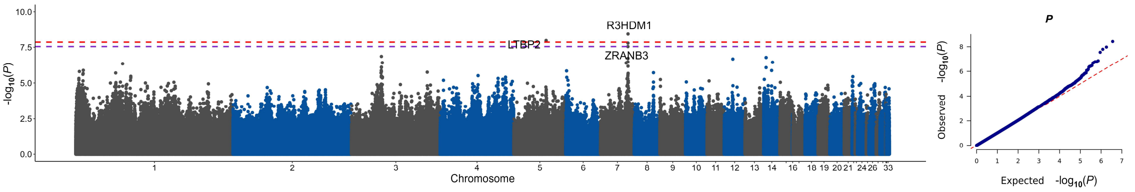

Supplementary Fig. S3. Manhattan and Q-Q plots of SNP-based GWAS for the sustained-TILI trait. The red dash line is the Bonferroni threshold and purple dash line is the 5% FDR threshold. SNPs passing the FDR threshold in the SNP-based GWAS were annotated for genes and labeled.

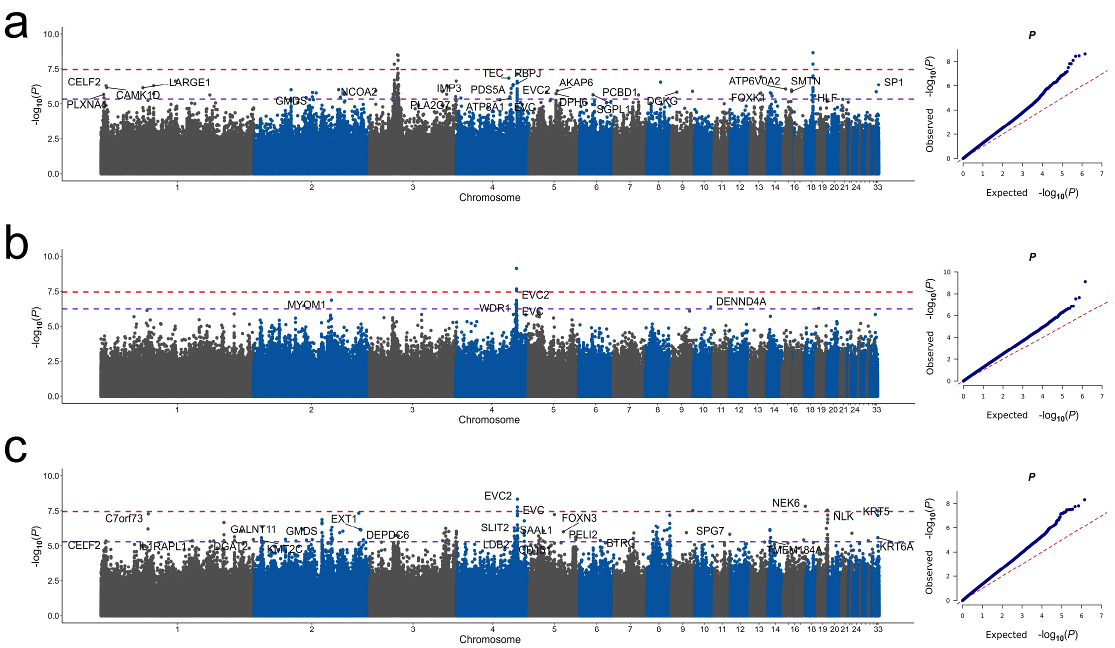

Supplementary Fig. S4. Genome-wide haplotype-based GWAS results for other traits with significant signals. a) Manhattan and Q-Q plot for the all-TILI trait. b) Manhattan and Q-Q plot for the all-WEV trait. c) Manhattan and Q-Q plot for the up-MILI trait. The red dash line is the Bonferroni threshold and purple dash line is the 5% FDR threshold. Haplotype-based GWAS identified additional significant loci of SNP-based GWAS in multiple egg-laying traits, demonstrating that haplotype-based GWAS outperforms SNP-based GWAS in power.

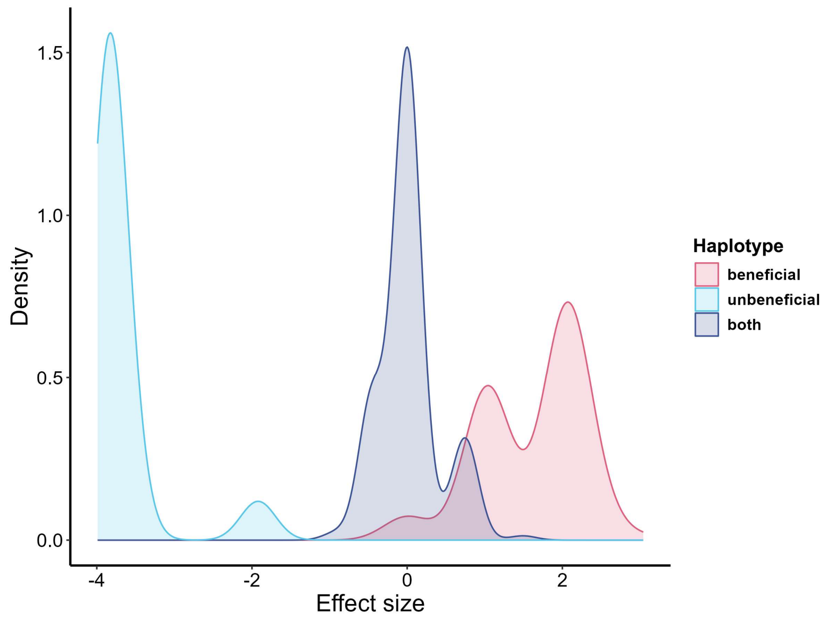

Supplementary Fig. S5. Density plot of effect size of haplotype alleles in significant haplotype blocks for the up-ECI trait. The X-axis is the estimated effect size of haplotypes and the Y-axis is the distribution density.

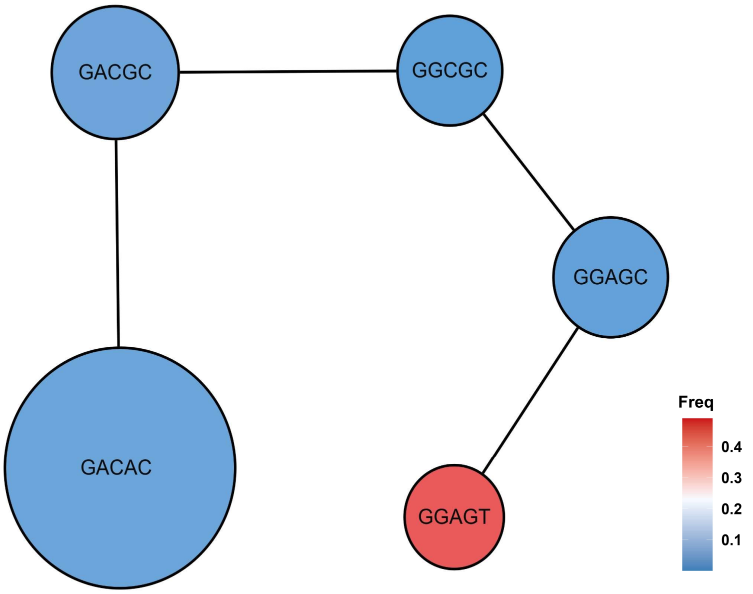

Supplementary Fig. S6. Effect size and frequency of haplotype alleles in haplotype block NO.247. The size of the circle indicates the effect size of haplotype alleles, and the color indicates its frequency in population. Same as in Fig. 4b, the dominant haplotype GGAGT had a low effect size, whereas the low-frequency haplotype GACAC had a high effect size.

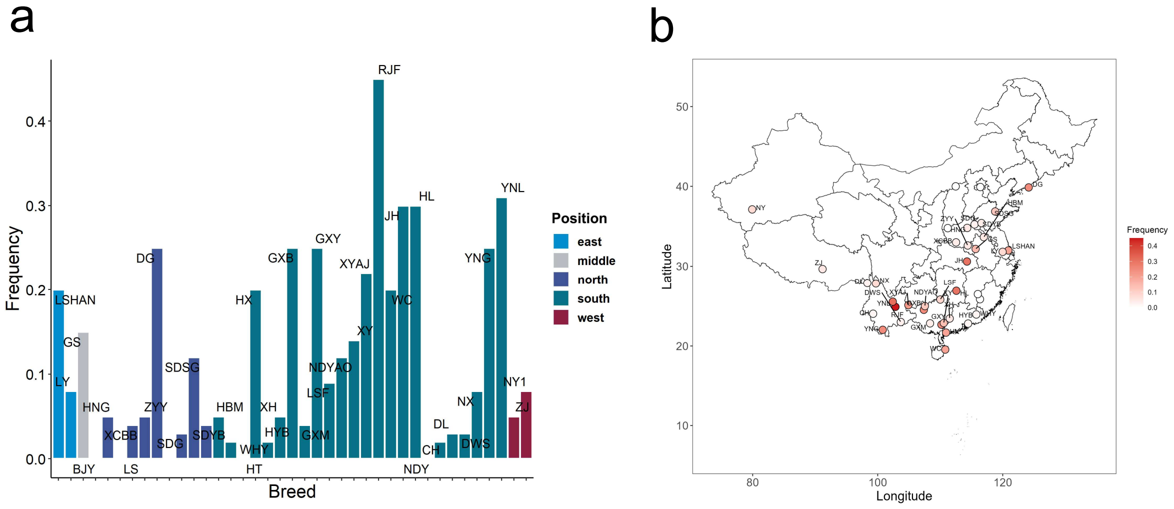

Supplementary Fig. S7. Frequency of beneficial haplotype allele for the up-ECI trait in Haplotype NO.247 in 39 Chinese local chicken populations. a) Among the chicken populations in the South, the highest frequency of beneficial haplotype allele GACAC was found in Red Jungle Fowl (RJF). Among the chicken populations in the North, the highest frequency was found in Dagu chicken (DG). b) Chicken populations exhibiting a higher frequency of the beneficial haplotype allele GACAC were predominantly located along the chicken migration routes from the South to the North in China.

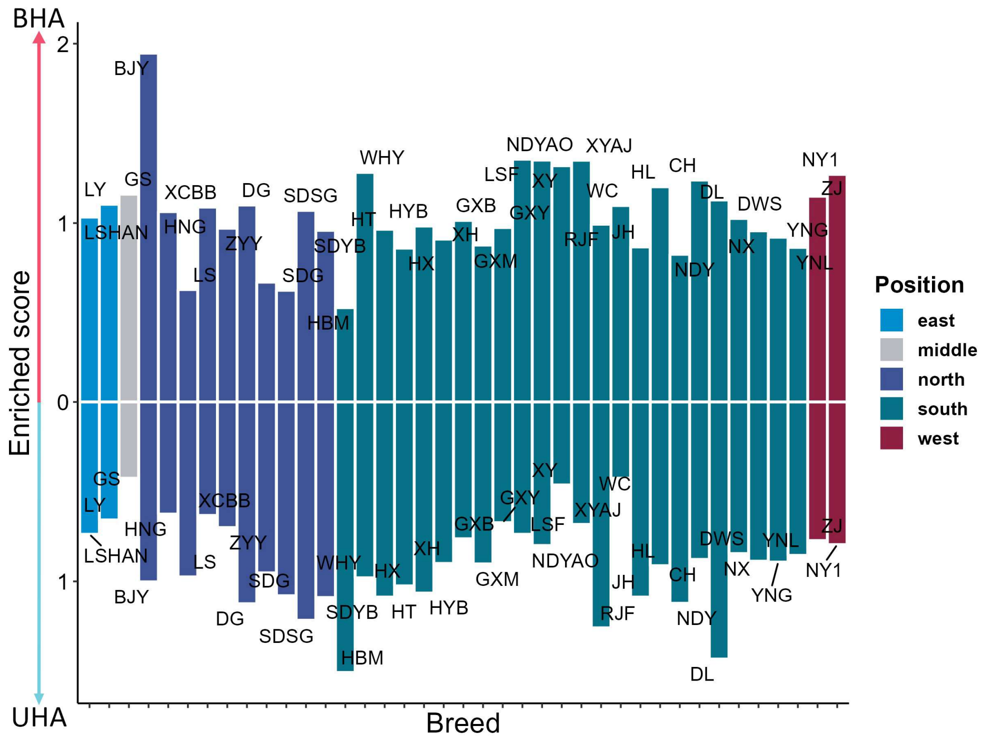

Supplementary Fig. S8: The enriched score for haplotype alleles in 39 local chicken populations for the all-WEV trait. Northern chickens had overall high enriched score to the haplotype allele of increasing variance, most notably for the Beijing You chicken (BJY). Southern chickens had overall high enriched score to the haplotype allele of decreasing variance, especially for by Huaibei Partridge chicken (HBM) and Dulong chicken (DL).

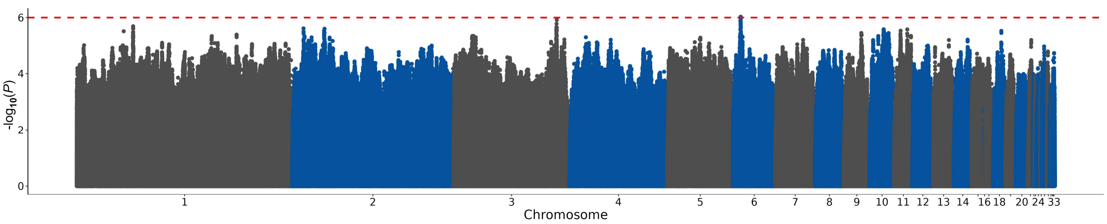

Supplementary Fig. S9: Genome-wide iHS signals for the chicken population. The red dashed line was the genome-wide Bonferroni threshold. iHS-transformed *P*-value shows only few SNPs passing the threshold on GGA6, suggesting that in the recent 1000 years, the chicken population was not strongly selected.

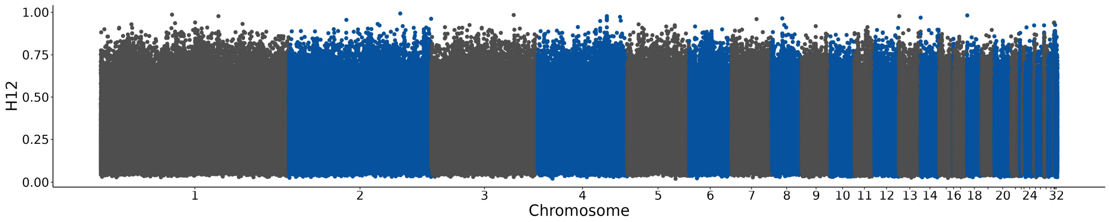

Supplementary Fig. S10: Genome-wide H12 signals for the chicken population. The H12 statistic indicates that almost no selection signal was found.

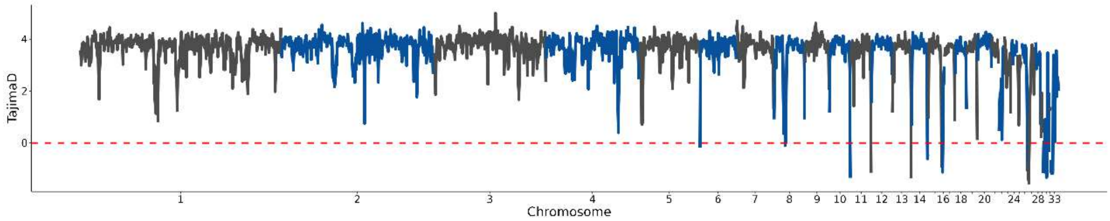

Supplementary Fig. S11: Genome-wide Tajima's D signal of the chicken population. Tajima's D of 0 indicates that the population has evolved exclusively according to genetic drift. Tajima's D greater than 0 indicates population expansion, selective sweep, or purifying selection. Tajima's D less than 0 indicates balancing selection, population bottleneck, or population subdivision. The majority of genome-wide Tajima's D values in the chicken population are positive, suggesting that the population may be experiencing a bottleneck effect.

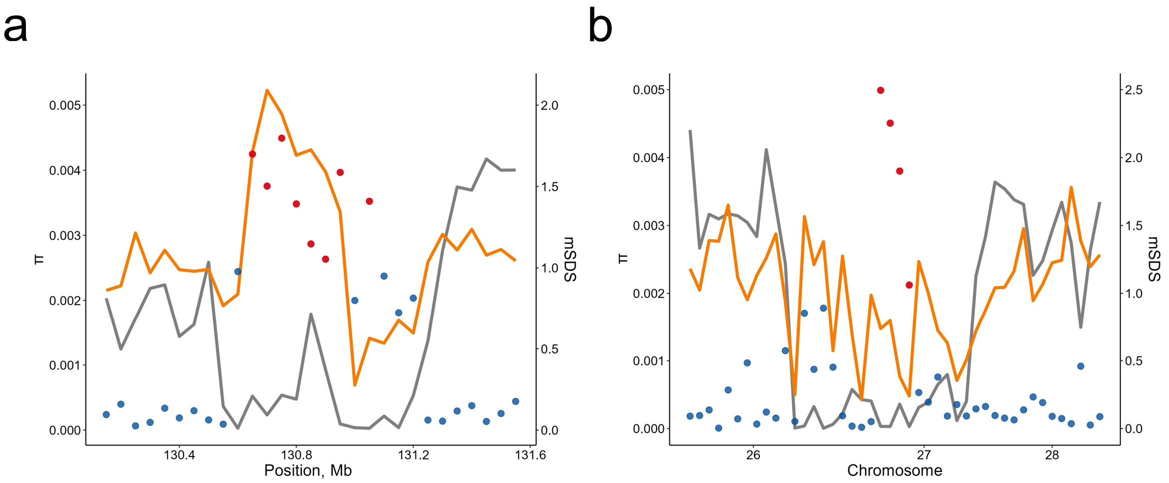

Supplementary Fig. S12: SDS, π, and iHS values for 50K significant blocks on high mSDS region. Scattered dots in the figure are mSDS values, with mSDS below 1 in blue and mSDS above 1 in red. The gray line is the π value, and the yellow line is the iHS value. a) Selection signals of high mSDS on the GGA2 region. Regions exhibiting high mSDS in this region also displayed correspondingly high iHS, suggesting these regions may have been subject to long-term selections. b) Selection signals of high mSDS on the GGA27 region. The region exhibiting high mSDS coincided with a low iHS, suggesting that this location has been under selection pressure for approximately the last 100 years. However, the low iHS indicates that artificial selection likely did not occur on more distant time scales.

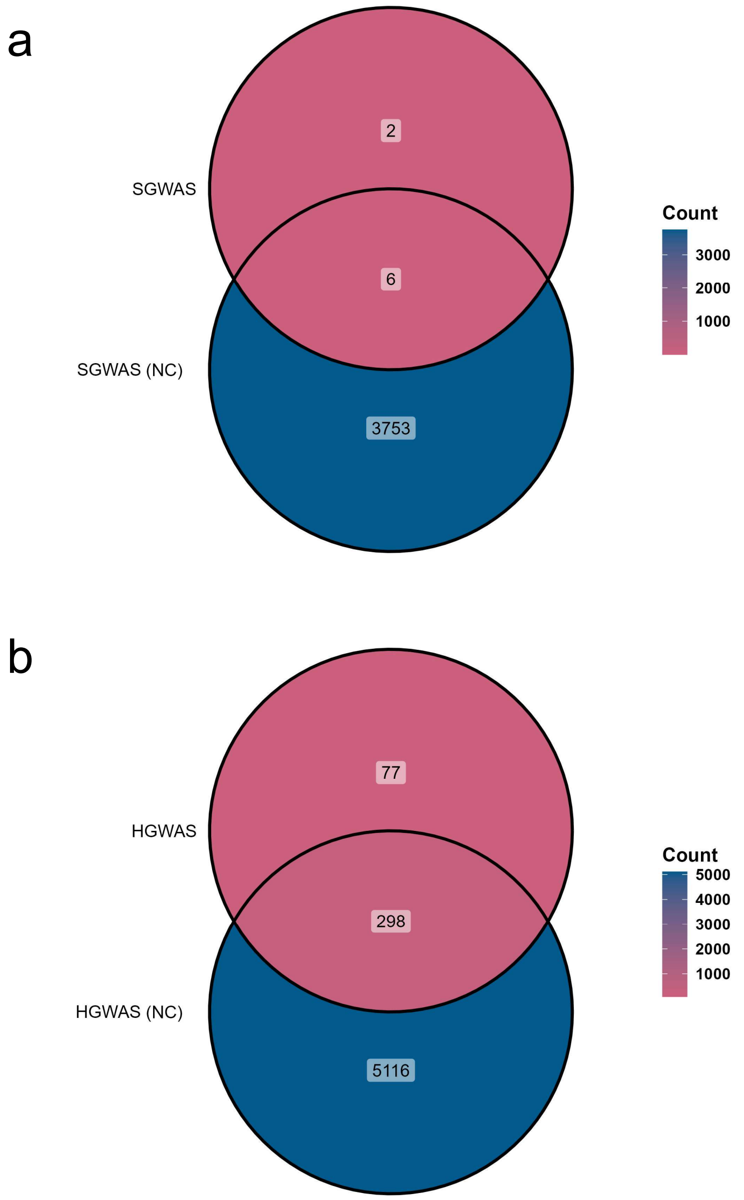

Supplementary Fig. S13. The overlap of SNP-based GWAS and haplotype-based GWAS between this study and our previous study (NC). a) Venn diagram for localized gene of SNP-based GWAS (SGWAS) between this study and our previous study, and b) Venn diagram for localized gene of haplotype-based GWAS (HGWAS) between this study and our previous study (NC). Localized genes in this study had high overlap with our previous study (NC).

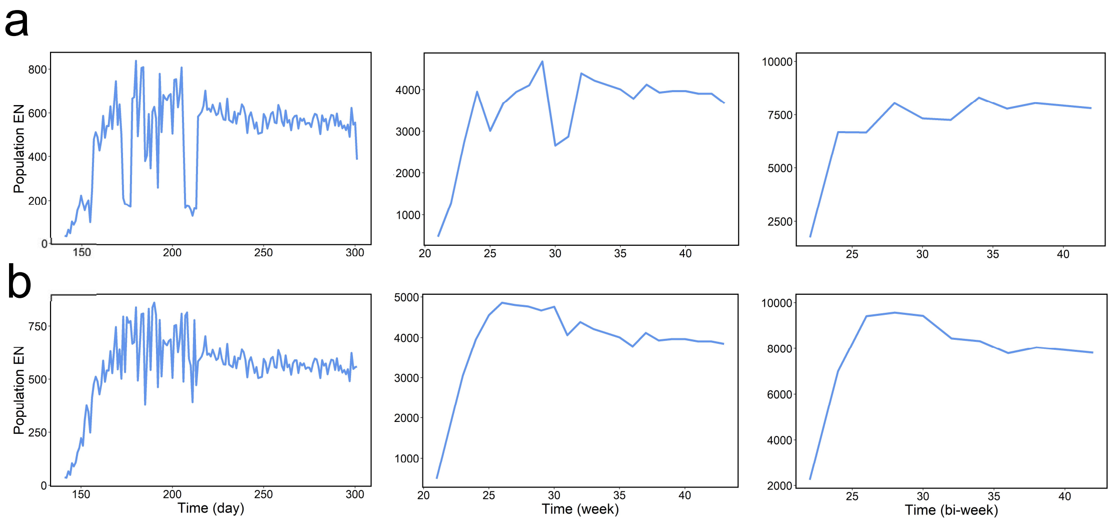

Supplementary Fig. S14. Egg-laying data before and after random forest imputing. (a) Total egg production daily, weekly, and bi-weekly for 21-43 weeks before random forest imputing, and b) Total egg production daily, weekly, and bi-weekly for 21-43 weeks after random forest imputing. As the time scale expanded, the trend in total egg production became progressively smoother, with initial gaps gradually disappearing. The egg-laying process clearly exhibited distinct periods of increase and decrease.

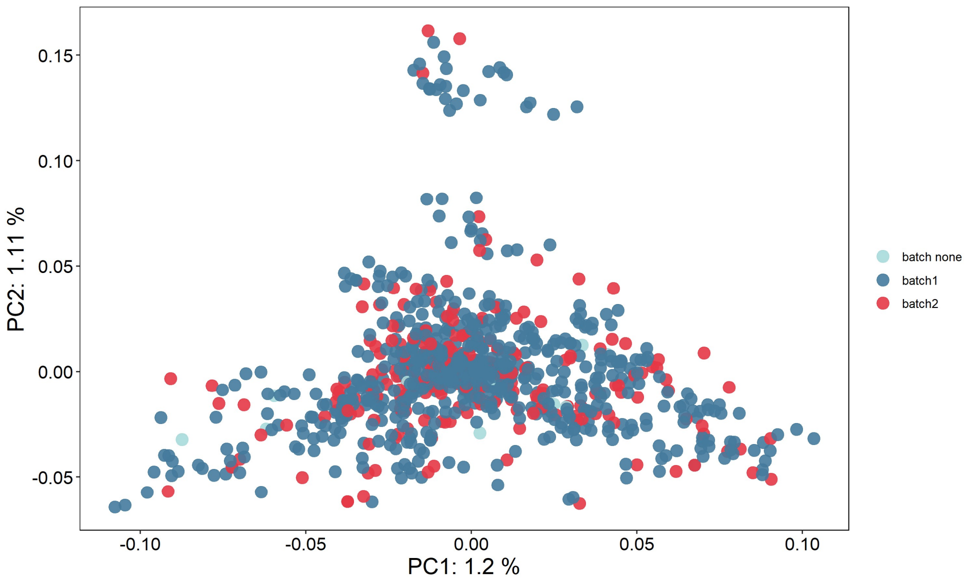

Supplementary Fig. S15: PCA projections of 888 chickens. Light blue scatter indicates individuals of unknown batch, dark blue indicates individuals of the first batch, and red indicates individuals of the second batch. PC1 explains 1.2% of the total variance, and there was no obvious stratification between batches.
